# Imbalanced Skeletal Muscle Mitochondrial Proteostasis Causes Bone Loss

**DOI:** 10.34133/research.0465

**Published:** 2024-08-30

**Authors:** Zhen Jin, Yan Mao, Qiqi Guo, Yujing Yin, Abdukahar Kiram, Danxia Zhou, Jing Yang, Zheng Zhou, Jiachen Xue, Zhenhua Feng, Zhen Liu, Yong Qiu, Tingting Fu, Zhenji Gan, Zezhang Zhu

**Affiliations:** ^1^ Division of Spine Surgery, Department of Orthopedic Surgery, Nanjing Drum Tower Hospital, Affiliated Hospital of Nanjing University Medical School, MOE Key Laboratory of Model Animal for Disease Study, Model Animal Research Center, Medical School of Nanjing University, Nanjing University, Nanjing, China.; ^2^ Division of Spine Surgery, Department of Orthopedic Surgery, Nanjing Drum Tower Hospital Clinical College of Nanjing Medical University, Nanjing, China.

## Abstract

Although microgravity has been implicated in osteoporosis, the precise molecular mechanism remains elusive. Here, we found that microgravity might induce mitochondrial protein buildup in skeletal muscle, alongside reduced levels of LONP1 protein. We revealed that disruptions in mitochondrial proteolysis, induced by the targeted skeletal muscle-specific deletion of the essential mitochondrial protease LONP1 or by the acute inducible deletion of muscle LONP1 in adult mice, cause reduced bone mass and compromised mechanical function. Moreover, the bone loss and weakness phenotypes were recapitulated in skeletal muscle-specific overexpressing ΔOTC mice, a known protein degraded by LONP1. Mechanistically, mitochondrial proteostasis imbalance triggered the mitochondrial unfolded protein response (UPR^mt^) in muscle, leading to an up-regulation of multiple myokines, including FGF21, which acts as a pro-osteoclastogenic factor. Surprisingly, this mitochondrial proteostasis stress influenced muscle–bone crosstalk independently of ATF4 in skeletal muscle. Furthermore, we established a marked association between serum FGF21 levels and bone health in humans. These findings emphasize the pivotal role of skeletal muscle mitochondrial proteostasis in responding to alterations in loading conditions and in coordinating UPR^mt^ to modulate bone metabolism.

## Introduction

Osteoporosis, a degenerative disorder associated with aging, manifests as reduced bone mass, micro-architectural degradation, and an increased susceptibility to hip fractures [1]. This condition stems from the dynamic nature of bone, which undergoes continuous remodeling orchestrated by osteoclasts and osteoblasts, responsible for bone resorption and formation, respectively [2]. Positioned proximately, bones and muscles work as a cohesive unit, where muscles apply load and bones serve as attachment sites. Exercise provides marked mechanical stimuli to the musculoskeletal system, leading to enhanced muscle function and bone health [3–5]. Conversely, the lack of this regulatory input due to disuse (such as during prolonged bed rest, exposure to microgravity, immobilization from a cast, or reduced physical activity) leads to conditions where muscles undergo catabolism, and bone experiences rapid resorption [6,7]. However, the potential molecular mechanism underlying muscle and bone loss due to microgravity remains unclear.

While the mechanical interplay between muscle and bone, it is evident that diminished mechanical loading, as seen in many muscle atrophies alone, is unlikely to fully explain the bone loss. Recent studies have realized the importance of muscle-derived biochemical factors, like myokines, in the regulation of bone mass [8]. For example, myostatin, pertaining to tumor growth factor-β (TGF-β) family, has been recognized as an inhibitor of bone mass [9]. Conversely, irisin, a cleaved product of fibronectin type III domain-containing 5 (FNDC5), is actively produced in skeletal muscle after exercise and exhibits dual roles as both a promoter and inhibitor of bone mass regulation, contingent upon its concentration and the hormonal environment [10,11]. Therefore, the molecular mechanisms governing the communication between muscle and bone are important for identifying potential therapies to maintain bone health.

Mitochondrial proteases are involved in the mechanisms of quality control, and these mechanisms target damaged or dysfunctional mitochondrial proteins for degradation. Dysregulation of mitochondrial proteostasis arises during cellular stress conditions and has been linked to the pathogenesis of numerous human diseases, including aging, metabolic disorders, cancer, and neurodegenerative diseases [12–15]. Roughly ^2^/_3_ of the 1,200 mitochondrial proteins reside in the matrix. The primary adenosine triphosphate (ATP)-dependent proteases, such as Lon protease homolog (LONP1) and Clp protease proteolytic subunit (CLPP), are emerging as central protein folding homeostasis control mechanism in mitochondria by clearing the non-assembled, misfolded, or damaged proteins [12–14,16]. The LONP1 protein resides on the mitochondria and constitutes the most conserved mitochondrial proteostasis from yeast to humans, and loss of LONP1 disturbs mitochondrial protein turnover, resulting in mitochondrial dysfunction [17–19].

Mitochondria have emerged as intriguing signaling hubs, not only interacting with various cellular compartments, such as the endoplasmic reticulum, to regulate cellular processes but also exerting influences on distant tissues, thereby impacting overall organismal health [20–22]. Recent evidence highlights the importance of imbalanced mitochondrial proteostasis in triggering mitochondrial-to-nuclear communication, termed the mitochondrial unfolded protein response (UPR^mt^), which leads to adaptive genomic reprogramming. Activation of UPR^mt^ can be advantageous as it allows for fine-tuning cellular metabolism and providing protection against pathogens [23–26]. Remarkably, in reaction to mitochondrial stress, skeletal muscle UPR^mt^ activated myokines such as fibroblast growth factor 21 (FGF21) and growth differentiation factor 15 (GDF15) have been identified as key mediators of distal organ communication in both mice and humans [27–30]. However, whether and how mitochondrial proteostasis in muscle regulates bone homeostasis remain unclear.

In this study, we investigated the consequences of imbalanced skeletal muscle mitochondrial proteostasis on bone health in the context of microgravity. Our findings suggest that microgravity can trigger the accumulation of mitochondrial proteins in skeletal muscle, concomitant with decreased levels of LONP1 protein. Subsequent investigations revealed that imbalanced skeletal muscle mitochondrial proteostasis caused by muscle LONP1 deficiency results in severe osteoporosis. Remarkably, these pathological features were faithfully recapitulated in mouse skeletal muscle-specific overexpressing mitochondrial-retained ΔOTC, a protein typically targeted for degradation by LONP1. Furthermore, we revealed that the perturbed mitochondrial proteostasis activated an unfolded protein response within skeletal muscle, known as UPR^mt^, which subsequently caused bone loss. Importantly, this bone loss appears to be mediated by the release of secreted factors originating from skeletal muscle in an ATF4-independent manner. A noteworthy discovery is the pivotal role played by FGF21, a myokine induced by UPR^mt^, in the context of imbalanced muscle mitochondrial proteostasis, influencing bone health in both murine and human contexts. Our study uncovers the importance of maintaining proper mitochondrial proteostasis in skeletal muscle to ensure the harmonious interplay between muscle and bone.

## Results

### The down-regulation of LONP1 correlates microgravity-induced mitochondrial protein accumulation in skeletal muscle

**Fig. 1.**
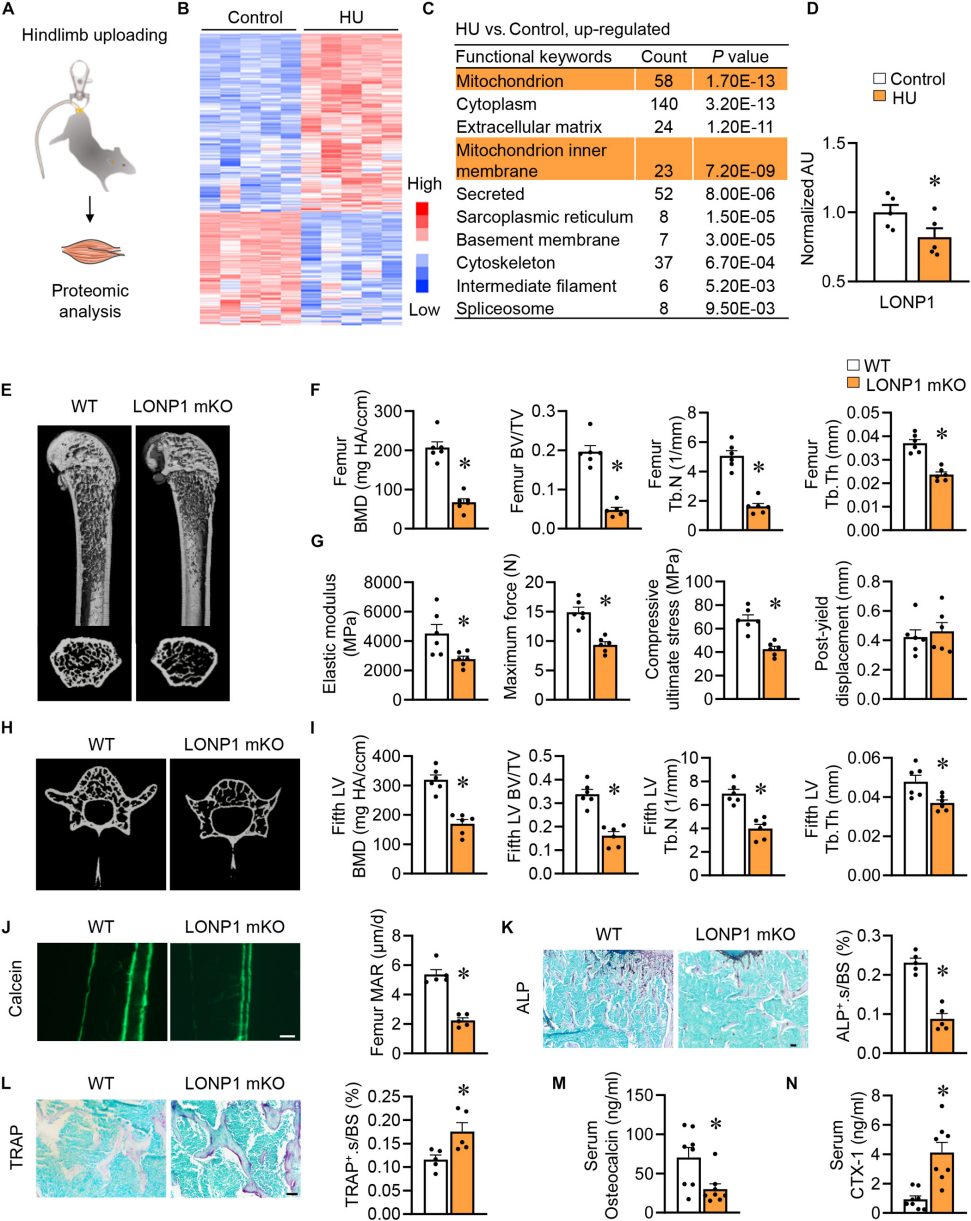
Skeletal muscle-specific ablation of LONP1 causes bone loss and mechanical impairments. (A to D) Scheme (A) for proteomic analysis using muscle samples from HU and control mice. Data were obtained from published dataset PXD041190. (B) Heatmap showing differentially regulated proteins in HU muscles (*n* = 5). (C) GO analysis from up-regulated proteins in HU muscles (*n* = 5). (D) Bar graphs for comparison of normalized protein expression of LONP1 in muscle tissues from HU muscles and controls (*n* = 5). (E to N) WT and LONP1 mKO male mice were harvested at 8 weeks old. (E and F) μCT pictures (E) and quantification (F) of trabecular bone parameters in the distal femur metaphysis (*n* = 6). (G) Bending test analysis of elastic modulus, maximum force, compressive ultimate stress, and post-yield displacement of femurs (*n* = 6). (H and I) μCT pictures (H) and quantification (I) of trabecular bone parameters in the fifth LVs (*n* = 6). (J) Pictures of calcein double labeling in femur sections (scale bar, 50 μm) and quantification of MAR (*n* = 5). (K) Images of ALP staining in femur sections (scale bar, 50 μm) and quantification of ALP^+^ cell surface per bone surface (ALP^+^.s/BS) in femurs (*n* = 5). (L) TRAP staining pictures in femur sections (scale bar, 50 μm) and quantification of TRAP^+^ cell surface per bone surface (TRAP^+^.s/BS) in femurs (*n* = 5). (M and N) Serum osteocalcin levels (M) and CTX-1 levels (N) (*n* = 8). Data are shown as the mean ± SEM. *P < 0.05 versus corresponding controls. *P* values were determined by an unpaired 2-tailed Student’s *t* test.

To delve into the molecular mechanism driving muscle and bone loss induced by microgravity, we utilized a proteomic dataset derived from skeletal muscle samples of a hindlimb unloading (HU) mice model (Fig. 1A) [31], a well-established model for studying osteoporosis and muscle atrophy resulting from mechanical unloading [32,33]. A comparison of the protein expression profiles between HU and control mice muscles unveiled 516 differentially expressed proteins (with a cutoff of 1.2-fold change and *Q* < 0.05), with 316 proteins up-regulated and 200 proteins down-regulated, respectively (Fig. 1B), indicating that muscle protein networks were substantially altered in muscles of HU mice. Gene ontology (GO)-based analysis revealed that the primarily up-regulated proteins in the HU muscles were localized within the mitochondria, especially mitochondrion inner membrane (Fig. 1C), suggesting the accumulation of mitochondrial protein. Specifically, LONP1, a central mitoprotease regulating mitochondrial proteostasis [12–14,16], exhibited decreased protein levels in alignment with increased mitochondrial protein content in HU muscles (Fig. 1D). This finding supports the hypothesis that microgravity may lead to mitochondrial protein accumulation in skeletal muscle and decreased LONP1 protein levels, both of which may have adverse effects on bone.

### Skeletal muscle-specific ablation of LONP1 causes bone loss and mechanical impairments

To determine whether disturbed skeletal muscle mitochondrial proteostasis impairs bone homeostasis, we took advantage of skeletal muscle-specific LONP1 knockout (LONP1 mKO) mice [34]. Initially, we assessed *Lonp1* expression levels in femurs of both wild-type (WT) and LONP1 mKO mice. Reverse transcription polymerase chain reaction (RT-PCR) analysis showed no marked transcriptional changes in *Lonp1* expression between the femurs of both mice, suggesting that HSA-Cre did not diffuse into the bone tissue (Fig. S1A). Subsequently, we conducted a comprehensive evaluation of bone phenotypes in LONP1 mKO mice. Micro-computed tomography (μCT) analysis of the femurs was performed, and this analysis revealed low trabecular bone volume (BV) and bone mineral density (BMD). The trabeculae from LONP1 mKO mice also showed decreased trabecular number (Tb.N) and trabecular thickness (Tb.Th) than WT littermates (Fig. 1E and F). Moreover, femoral bending tests indicated compromised mechanical integrity in LONP1 mKO mice, as evidenced by decreased elastic modulus, maximum force, and compressive ultimate stress (Fig. 1G). Similar trends were observed in the fifth lumbar vertebral bodies (fifth LVs) of LONP1 mKO mice, with notable reductions in BV/TV (bone volume/total volume), BMD, Tb.N, and Tb.Th relative to WT controls (Fig. 1H and I). These findings remained consistent in female mice with muscle-specific LONP1 disruption, demonstrating marked reductions in trabecular mass and microstructure in both femurs and the fifth LVs (Fig. S1B to E). Additionally, double labeling of calcein indicated a lower mineral apposition rate (MAR) in LONP1 mKO mice (Fig. 1J). Furthermore, ALP (alkaline phosphatase) staining unveiled a substantial reduction in the surface area of osteoblasts in trabecular (Fig. 1K), while TRAP (tartrate-resistant acid phosphatase) staining indicated a remarkable increase in the surface area of osteoclasts within trabecular region (Fig. 1L). Biochemical tests of blood plasma showed reduced levels of osteocalcin (a biomarker of bone formation) and elevated levels of the C-terminal telopeptides of type I collagen (CTX-1, a biomarker of bone resorption) (Fig. 1M and N).

A high-fat diet (HFD) is recognized as an environmental factor linked to accelerated bone loss during skeletal aging [35]. To further underscore the importance of muscle LONP1 in bone metabolism regulation, we examined whether the absence of muscle LONP1 influenced bone health under HFD conditions. Notably, HFD-fed LONP1 mKO mice displayed a marked reduction in bone mass, as evidenced by a decrease in trabecular mass and microstructure in the femur relative to WT controls (Fig. S2A and B). Moreover, femoral bending tests revealed a decline in elastic modulus, maximum force, and compressive ultimate stress in HFD-treated LONP1 mKO mice (Fig. S2C), further emphasizing the detrimental effect on mechanical integrity. Importantly, the fifth LVs of the HFD-treated LONP1 mKO mice also exhibited a lower BV, BMD, Tb.N, and Tb.Th (Fig. S2D and E). In line with our observations in mice fed a standard chow diet (CD), HFD-fed LONP1 mKO mice displayed decreased levels of osteocalcin and increased levels of CTX-1 (Fig. S1F and G). These results underscore the importance of skeletal muscle LONP1 in preserving bone mass and function, regardless of dietary conditions, including those associated with an HFD.

### The sudden loss of LONP1 in mature muscles results in bone loss

**Fig. 2.**
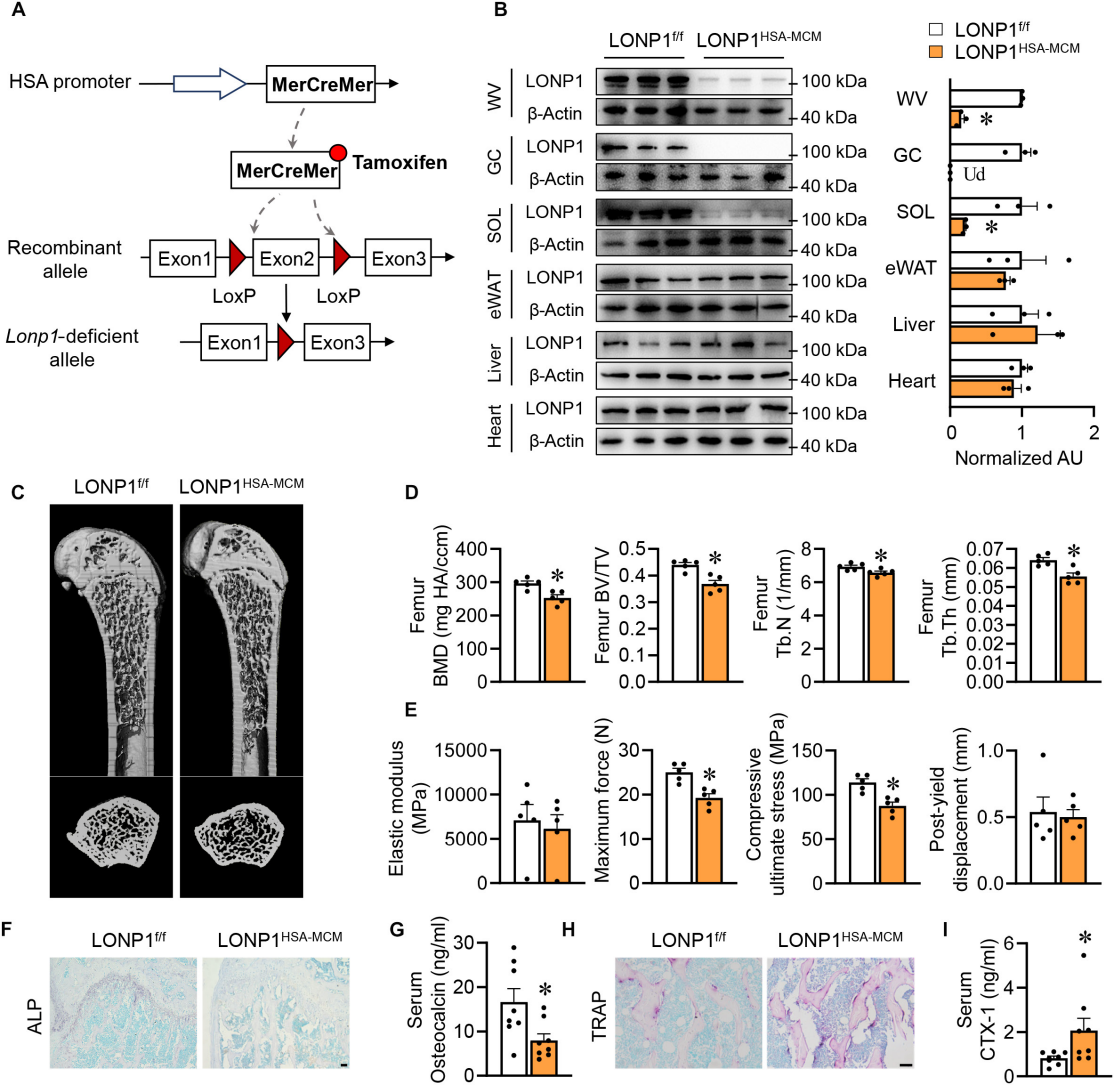
The sudden loss of LONP1 in mature muscles results in bone loss. (A) Diagrammatic drawing demonstrating the generation of tamoxifen-inducible muscle-specific deletion of *Lonp1* (LONP1^HSA-MCM^). (B to I) LONP1^f/f^ and LONP1^HSA-MCM^ male mice were fed CD and injected by tamoxifen at 6 weeks old and harvested at 15 weeks old. (B) Representative quantification of LONP1 protein expression in skeletal muscles, eWAT, liver, and heart from LONP1^f/f^ and LONP1^HSA-MCM^ male mice. Quantification of the LONP1/β-actin ratio was determined by ImageJ (*n* = 3). (C and D) μCT pictures (C) and quantification (D) of trabecular bone parameters in the distal femur metaphysis (*n* = 5). (E) Bending test analysis of elastic modulus, maximum force, compressive ultimate stress, and post-yield displacement of femurs from LONP1^f/f^ and LONP1^HSA-MCM^ mice (*n* = 5). (F and H) Pictures of ALP staining and TRAP staining in femur sections (scale bar, 50 μm). (G and I) Serum osteocalcin levels (G) and CTX-1 levels (I) of LONP1^f/f^ and LONP1^HSA-MCM^ mice (*n* = 8). Data are shown as the mean ± SEM. *P < 0.05 versus corresponding controls. *P* values were determined by an unpaired 2-tailed Student’s *t* test. Ud, undetectable.

To mimic the sudden loss of gravity during space flight or long-term bed rest, we generated tamoxifen-inducible myofiber LONP1 knockout mice (LONP1^HSA-MCM^) by breeding LONP1^f/f^ mice with HSA-MCM mice (Fig. 2A) [20]. Notably, tamoxifen treatment resulted in a notable decrease in LONP1 protein levels within the white vastus lateralis (WV), gastrocnemius (GC), and soleus (Sol) muscles from LONP1^HSA-MCM^ mice, while levels remained unchanged in epididymal white adipose tissue (eWAT), liver, and heart (Fig. 2B). Similar to the muscle atrophy induced by microgravity, the sudden loss of muscle LONP1 decreased the weight of GC and tibialis anterior (TA) muscle (Fig. S4A). Quantitative analysis of muscle fiber size distribution showed a notable decrease in myofiber size in LONP1^HSA-MCM^ mice (Fig. S4B). Simultaneously, we investigated the impact of acute muscle LONP1 loss on bone. The μCT analysis revealed a substantial decrease in bone mass, as indicated by declines in femoral trabecular BMD, volume, number, and thickness of LONP1^HSA-MCM^ mice compared to their LONP1^f/f^ littermates (Fig. 2C and D). Additionally, femoral bending tests demonstrated a reduction in maximum force and compressive ultimate stress (Fig. 2E). Similarly, the LONP1^HSA-MCM^ mice also showed lower bone mass in the fifth LV (Fig. S3A and B). Furthermore, ALP staining and plasma osteocalcin tests revealed decreased bone formation in LONP1^HSA-MCM^ mice (Fig. 2F and G), while TRAP staining and plasma CTX-1 tests indicated markedly increased bone resorption in LONP1^HSA-MCM^ mice (Fig. 2H and I). Additionally, HFD-fed LONP1^HSA-MCM^ mice exhibited a profound reduction in bone mass, including lower trabecular BV, number, thickness, and BMD than their littermate (Fig. S4C and D). Moreover, femoral bending tests revealed a decline in elastic modulus, maximum force, and compressive ultimate stress in HFD-fed LONP1^HSA-MCM^ mice (Fig. S4E). The fifth LVs of the HFD-fed LONP1^HSA-MCM^ mice also displayed decreased bone mass (Fig. S4F and G). Thus, mature skeletal muscle continuously participates in tuning bone metabolism, and this interaction is not confined to developmental stages.

### Acute LONP1 deletion in skeletal muscle impacts diet-induced obesity and insulin resistance

We have demonstrated the essential role of muscle LONP1 in preserving systemic metabolic equilibrium [36], as well as sustaining muscle vitality [34] and overall bone health. Next, we evaluated the possible effect of acute muscle LONP1 deletion in the context of HFD-induced obesity. Before tamoxifen treatment, no difference of body weight between LONP1^f/f^ and LONP1^HSA-MCM^ mice was observed. Following this, mice were subjected to tamoxifen treatment to eliminate skeletal muscle LONP1, after which they were maintained on HFD for 20 weeks. Notably, the HFD-fed LONP1^HSA-MCM^ mice exhibited markedly lower body weight than the HFD-fed LONP1^f/f^ littermates after 18 weeks (Fig. S5A). HFD-fed LONP1^HSA-MCM^ mice exhibited enhanced glucose tolerance and heightened insulin sensitivity (Fig. S5B and C). Hematoxylin and eosin (H&E) and Oil Red O staining of liver sections revealed improved hepatic steatosis in HFD-fed LONP1^HSA-MCM^ mice (Fig. S5D). These data imply that acute muscle LONP1 loss can also impact systemic metabolism.

### Skeletal muscle-specific overexpression of mitochondrial-retained ΔOTC protein induces bone loss

**Fig. 3.**
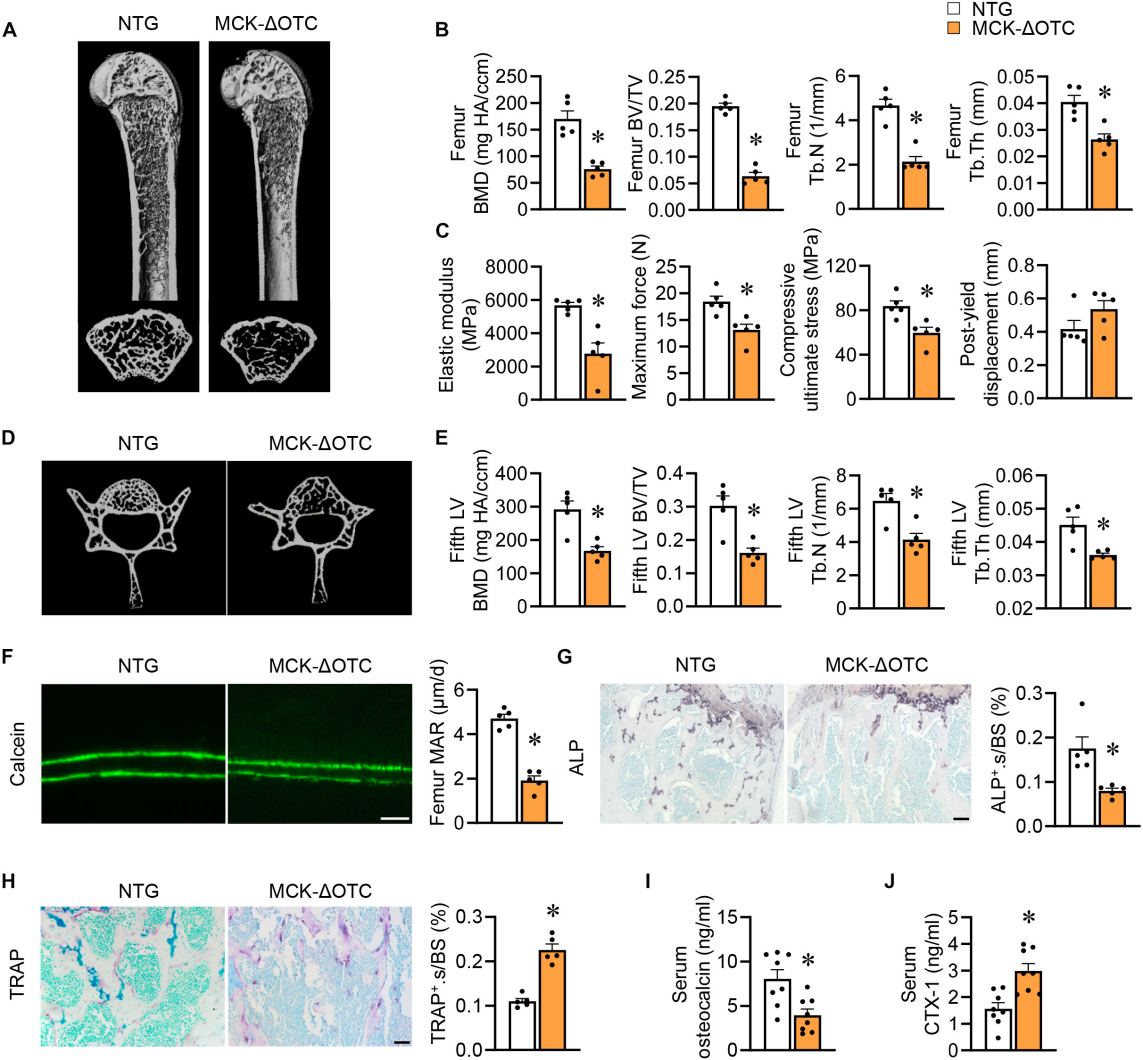
Skeletal muscle-specific overexpression of mitochondrial-retained ΔOTC protein induces bone loss. (A to J) NTG and MCK-ΔOTC male mice were harvested at 8 weeks old. (A and B) μCT pictures (A) and quantification (B) of trabecular bone parameters in the distal femur metaphysis (*n* = 5). (C) Bending test analysis of elastic modulus, maximum force, compressive ultimate stress, and post-yield displacement of femurs from NTG and MCK-ΔOTC mKO. (D and E) μCT pictures (D) and quantification (E) of trabecular bone parameters in the fifth LVs (*n* = 5). (F) Pictures of calcein double labeling in femur sections (scale bar, 50 μm) and quantitative analysis of MAR (*n* = 5). (G) Images of ALP staining in femur sections (scale bar, 50 μm) and quantification of ALP^+^.s/BS (*n* = 5). (H) Pictures of TRAP staining in femur sections (scale bar, 50 μm) and quantification of TRAP^+^.s/BS (*n* = 5). (I and J) Serum osteocalcin levels (I) and CTX-1 levels (J) (*n* = 8). Data are shown as the mean ± SEM. *P < 0.05 versus corresponding controls. *P* values were determined by an unpaired 2-tailed Student’s *t* test.

Next, we postulated that an overload of unfolded proteins within mitochondria, surpassing the capacity of LONP1 in skeletal muscle, could have analogous effects on bone health. To test this hypothesis, we turned to a well-established mice model for investigating mitochondrial proteostasis unbalance and UPR^mt^ [16,26,34,36,37]: transgenic mice that selectively overexpress a deletion mutant of a LONP1 substrate, ornithine transcarbamylase, exclusively in skeletal muscle (MCK-ΔOTC). As expected, a broad range of UPR^mt^ genes (e.g., *Atf4*, *Atf5*, *Chac1*, *Dnajb9*, *Rhbdd1*, *Herpud1*, *Ddit3*, *Wfs1*, *Hspd1*, and *Hspa9*) were activated in MCK-ΔOTC muscles, as confirmed by RT-PCR studies (Fig. S6A). Consistent with observations in LONP1 mKO mice, the overexpression of ΔOTC in skeletal muscle results in a remarkable decline in bone mass, observed in both male and female mice, as evidenced by μCT analysis of the proximal femur (Fig. 3A and Fig. S6B). Parameters associated with trabecular bone, including mineral density, volume, number, and thickness, were all decreased in relation to the nontransgenic (NTG) mice (Fig. 3B and Fig. S6C). Furthermore, MCK-ΔOTC mice exhibited declined elastic modulus, maximum force, and compressive ultimate stress (Fig. 3C). These phenotypes were similarly mirrored in the fifth LVs, with both male and female MCK-ΔOTC mice displaying decreased trabecular bone mass and structure (Fig. 3D and E and Fig. S6D and E). Histomorphometry and serum bone turnover marker analysis revealed a decrease in MAR, osteoblast surface, and serum osteocalcin levels due to ΔOTC overexpression (Fig. 3F, G, and I), alongside an elevation in osteoclasts count and serum CTX-1 (Fig. 3H and J). Collectively, these findings illustrate that overexpression of mitochondrial-retained ΔOTC protein in skeletal muscle is capable of inducing bone loss and mechanical impairment.

### Mitochondrial proteostasis stress elicits mitochondrial UPR and myokine expression in skeletal muscle

**Fig. 4.**
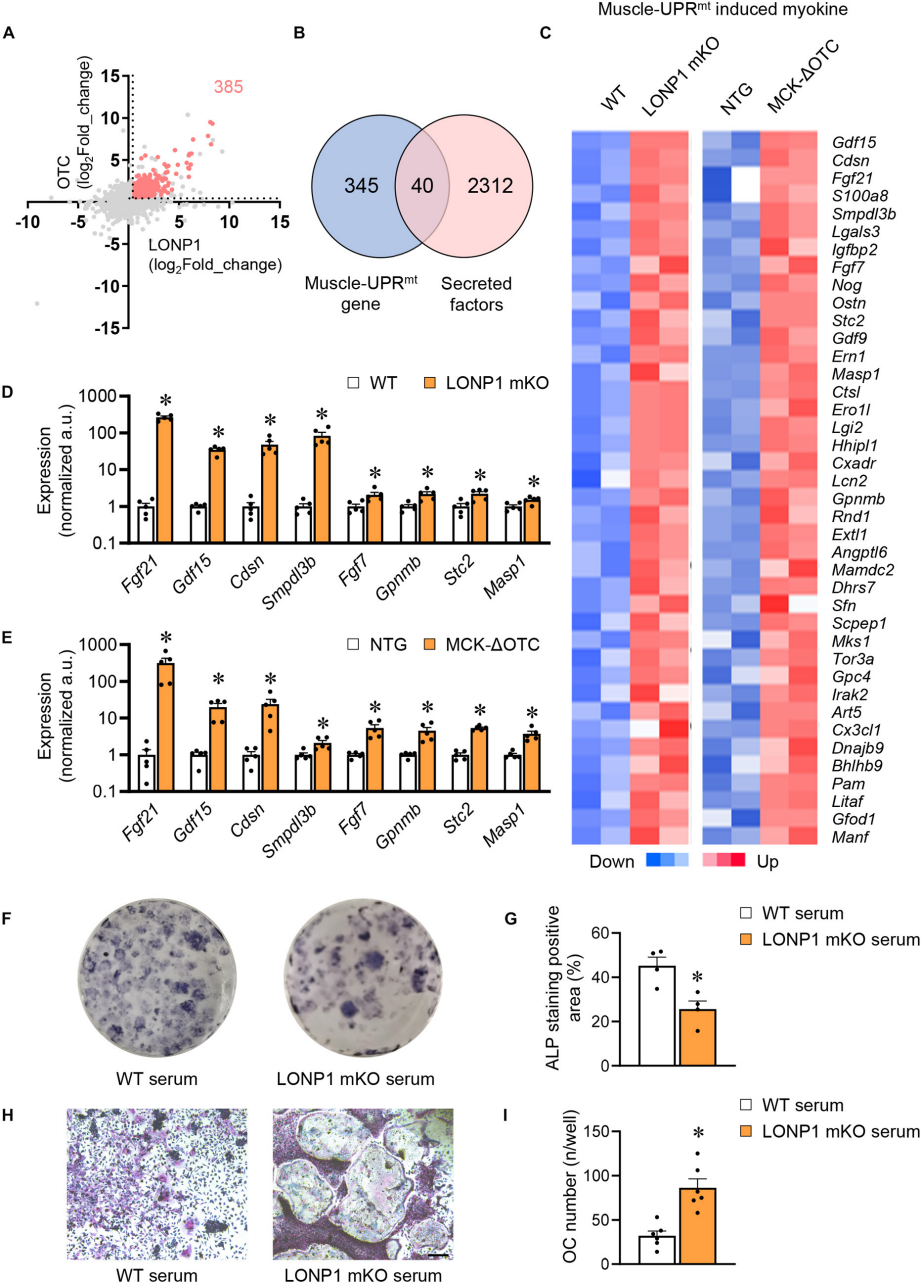
Mitochondrial proteostasis stress elicits mitochondrial UPR and myokine expression in skeletal muscle. (A) Bubble diagram showing fold changes for dataset (GSE192991) generated from the GC of MCK-ΔOTC mice compared to littermate control (NTG) mice versus fold changes for dataset (GSE166071) generated from the GC of LONP1-mKO mice compared to WT mice. Both up-regulated genes in the 2 groups are represented by pink dots. (B) Venn diagram exhibiting an overlap between secreted factors and both significantly up-regulated genes in LONP1-mKO and MCK-ΔOTC mice. (C) Heatmap analysis of muscle-UPR^mt^ myokine genes up-regulated in LONP1-mKO or MCK-ΔOTC mice compared to littermate controls (*n* = 2). (D) Gene expression of muscle-UPR^mt^ myokines in GC muscles in WT and LONP1 mKO mice (*n* = 5). (E) Gene expression of muscle-UPR^mt^ myokines in GC muscles in NTG and MCK-ΔOTC mice (*n* = 5). (F and G) ALP staining images (F) and quantification (G) of osteoblasts administered with the serum of WT and LONP1 mKO mice (*n* = 4). (H and I) TRAP staining images (H) and quantification (I) of osteoclasts administered with the serum of WT and LONP1 mKO mice (scale bar, 100 μm; *n* = 3). Data are shown as the mean ± SEM. *P < 0.05 versus corresponding controls. *P* values were determined by an unpaired 2-tailed Student’s *t* test.

In our prior research, we clarified that mitochondrial proteostasis stress is adequate to trigger UPR^mt^ in skeletal muscle [36]. To further explore the connection between muscle-UPR^mt^ and bone metabolism, we conducted whole-genome gene expression profiling experiments in GC of LONP1 mKO and MCK-ΔOTC mice (GSE166071 and GSE192991), identifying a common set of 385 up-regulated genes (fold change > 1.5 and *P* < 0.05, red dots) across both skeletal muscle models, which we termed the muscle-UPR^mt^ gene signature (Fig. 4A). This discovery prompted an exploration into myokines, signaling molecules released by muscle tissue, and their role in inter-organ communication. We found that 40 secreted factors were up-regulated in both LONP1 mKO and ΔOTC overexpression muscles (Fig. 4B and C and Table S1), suggesting that imbalanced mitochondrial proteostasis in muscles could activate secretory signals for inter-organ communication. RT-PCR validation studies confirmed the up-regulation of genes linked to UPR^mt^ and secreted myokines (e.g., *Fgf21*, *Gdf15*, *Cdsn*, *Smpdl3b*, *Fgf7*, *Gpnmb*, *Stc2*, and *Masp1*) in both LONP1 mKO and MCK-ΔOTC muscles (Fig. 4D and E). This discovery strengthens the hypothesis that the UPR^mt^-mediated signaling pathway in skeletal muscle potentially generates secretory molecules influencing bone health.

**Fig. 5.**
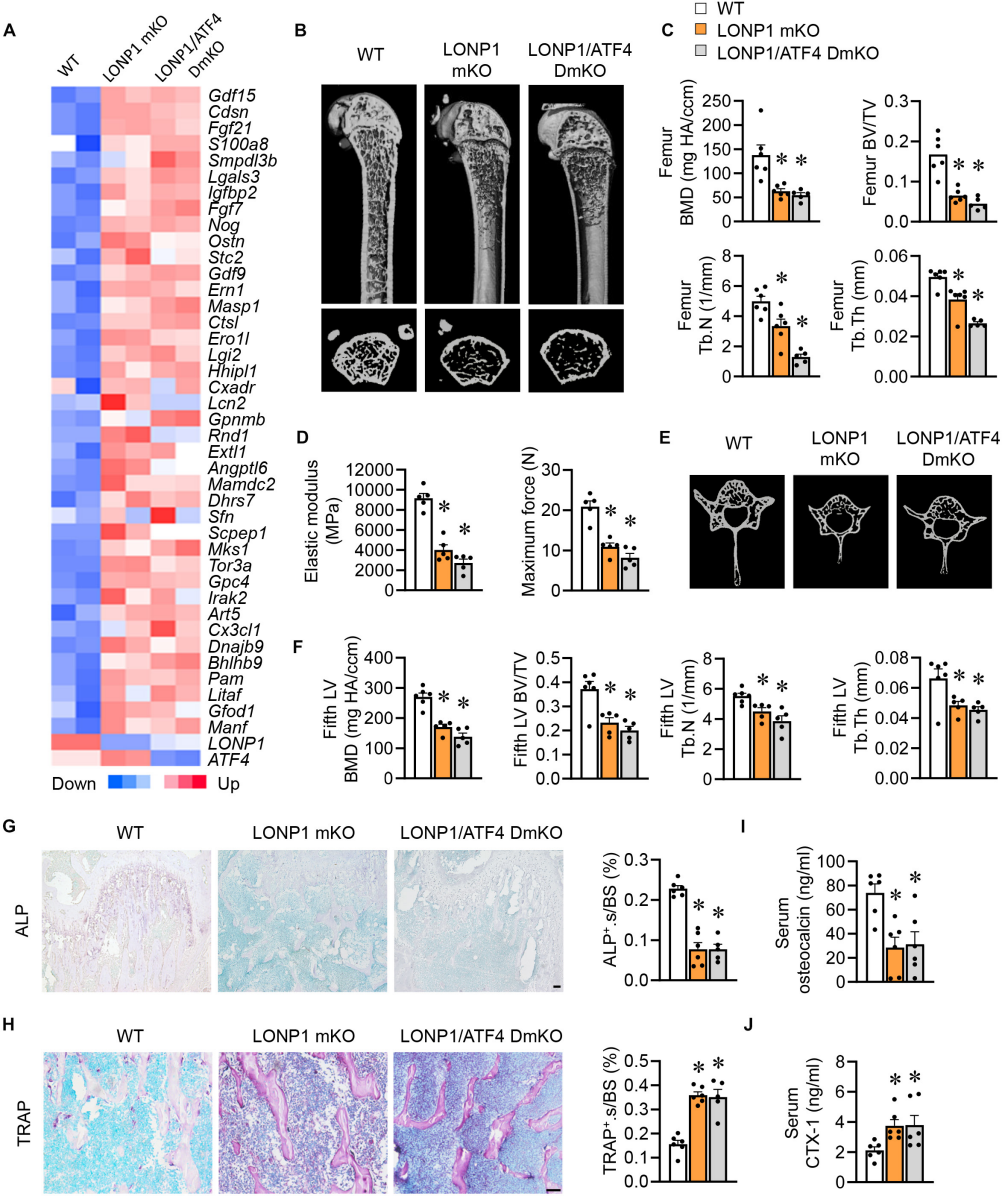
Skeletal muscle mitochondrial proteostasis stress-induced bone loss is independent of ATF4. (A) Heatmap analysis of muscle-UPR^mt^ myokines and *Lonp1* or *Atf4* genes in indicated mice (GSE192990; *n* = 2). (B to J) WT, LONP1 mKO, and LONP1/ATF4 DmKO male mice were harvested at 8 weeks old. (B and C) μCT pictures (B) and quantification (C) of trabecular bone parameters in the distal femur metaphysis (*n* = 5 to 6). (D) Bending test analysis of elastic modulus, maximum force, compressive ultimate stress, and post-yield displacement of femurs (*n* = 5). (E and F) μCT pictures (E) and quantification (F) of trabecular bone parameters in the fifth LVs (*n* = 5 to 6). (G) Pictures of ALP staining in femur sections (scale bar, 50 μm) and quantification of ALP^+^.s/BS (*n* = 5). (H) Pictures of TRAP staining in femur sections (scale bar, 50 μm) and quantification of TRAP^+^.s/BS (*n* = 5). (I and J) Serum osteocalcin levels (I) and CTX-1 levels (J) (*n* = 8). Data are shown as the mean ± SEM. *P < 0.05 versus corresponding controls. *P* values were determined by one-way ANOVA.

To investigate this prospect, we collected serum from WT and LONP1 mKO mice, examining their impact on osteoblast and osteoclast differentiation. Bone marrow cells (BMCs) were separated and differentiated into osteoblasts or osteoclasts following the published protocol. Bone marrow-derived mesenchymal stem cells (BM-MSCs) from WT mice were separated and differentiated into osteoblasts. The osteogenic differentiation effect of serum from LONP1mKO mice was attenuated to that of WT mice, as indicated by the reduced area of ALP-positive osteoblast (Fig. 4F and G). Additionally, we treated cultured bone marrow-derived macrophages (BMDMs) with serum from WT and LONP1 mKO mice, along with M-CSF (macrophage colony-stimulating factor) and RANKL (receptor activator of nuclear factor-κB ligand) stimulation for 5 d to induce osteoclast formation. Subsequently, TRAP staining was performed to assess osteoclast activity. As illustrated in Fig. 5H, serum from LONP1 mKO mice markedly enhanced RANKL-induced osteoclast differentiation (Fig. 4H and I). These results collectively suggest that mitochondrial proteostasis stress prompts muscle cells to secrete factors directly impacting osteogenesis and promoting osteoclastogenic activity, thus recapitulating the observed in vivo low bone mass in LONP1 mKO mice.

### Skeletal muscle mitochondrial proteostasis stress-induced bone loss is independent of ATF4

Activating transcription factor 4 (ATF4) has previously been linked to the regulation of mammalian UPR^mt^ [38,39]. Therefore, we explored whether the muscle ATF4 plays a role in mitochondrial proteostasis stress and bone homeostasis. To explore the impact of muscle ATF4 in the context of LONP1 mKO mice, we generated double knockout mice with LONP1 and ATF4 deficiencies (LONP1/ATF4 DmKO) by crossing ATF4^f/f^ mice with LONP1 mKO mice. Examining the effect of ATF4 deletion on bone turnover within LONP1 mKO, we conducted transcriptome analysis of muscles from WT, LONP1 mKO, and LONP1/ATF4 DmKO mice (GSE192990). Intriguingly, we observed that the expression of a majority of secreted factors in skeletal muscles induced by LONP1 deficiency remained unaffected by ATF4’s absence (Fig. 5A). Furthermore, μCT analysis of LONP1/ATF4 DmKO mice femurs did not demonstrate any improvement in trabecular BV, density, number, and thickness compared to LONP1 mKO mice (Fig. 5B and C). Similarly, femoral bending tests demonstrated that LONP1/ATF4 DmKO mice’s mechanical function remained impaired, akin to LONP1 mKO mice (Fig. 5D and Fig. S7A). Moreover, no marked differences were observed in trabecular bone mass and microstructure in the fifth LVs of LONP1/ATF4 DmKO mice in relation to LONP1 mKO mice (Fig. 5E and F). Additionally, histomorphometry and serum bone turnover marker analysis were revealed. Deletion of ATF4 did not affect bone formation in mice lacking skeletal muscle LONP1 (Fig. 5G and I), alongside no difference in reabsorption (Fig. 5H and J). Collectively, these findings indicate that muscle mitochondrial proteostasis stress-mediated bone loss occurs independently of ATF4.

### ATF4-independent regulation of FGF21 is linked to bone metabolism

**Fig. 6.**
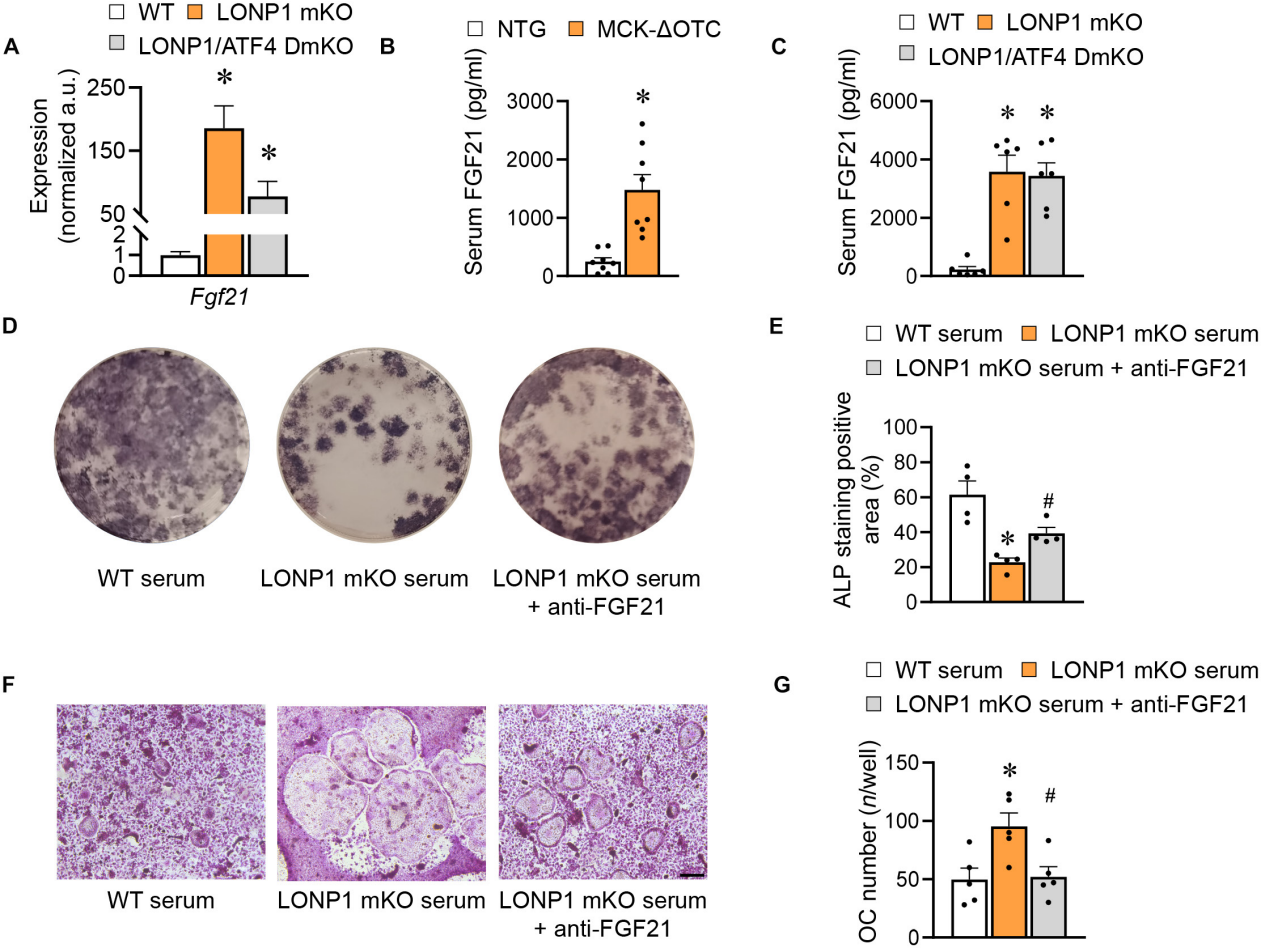
ATF4-independent regulation of FGF21 is linked to bone metabolism in mice and humans. (A) Relative mRNA expression of *Fgf21* in WT, LONP1 mKO, and LONP1/ATF4 DmKO mice (*n* = 5). (B) Serum FGF21 levels detected by ELISA kit (Proteintech, KE10042) in NTG and MCK-ΔOTC mice (*n* = 8). (C) Serum FGF21 levels detected by ELISA kit (Proteintech, KE10042) in WT, LONP1 mKO, and LONP1/ATF4 DmKO mice (*n* = 6). (D and E) ALP staining pictures (D) and quantification (E) of osteoblasts administered with the serum of WT and LONP1 mKO mice or combined with an FGF21-blocking antibody [anti-insulin-like growth factor 2 (IGF2), 1 μg/ml; *n* = 4]. (F and G) TRAP staining images (F) and quantification (G) of osteoclasts administered with the serum of WT and LONP1 mKO mice or combined with an FGF21-blocking antibody (anti-IGF2, 1 μg/ml; scale bar, 100 μm; *n* = 3). Data are shown as the mean ± SEM. *P < 0.05 versus corresponding controls. *P* values were determined by one-way ANOVA.

Next, we turned our attention to ATF4-independent myokines, which could potentially mediate communication between UPR^mt^-stressed skeletal muscle and bone. One particularly intriguing factor of interest is FGF21, a known marker of mitochondrial stress, which exhibited the most prominent up-regulation among the secreted factors in muscles of LONP1 mKO and MCK-ΔOTC mice (Fig. 4D and E). Recent studies have shown that FGF21 can promote bone resorption and led to bone loss [40,41]. To delve deeper into this observation, we examined the mRNA levels of FGF21 and observed that FGF21 remained markedly elevated, approximately 80-fold, in LONP1/ATF4 DmKO muscles compared with WT controls (Fig. 6A). Furthermore, we observed a noteworthy rise of serum FGF21 levels in both LONP1 mKO and MCK-ΔOTC mice (Fig. 6B and Fig. S7B). The elevated secreted FGF21 levels remained unaffected despite ATF4 deletion (Fig. 6C), suggesting that ATF4-independent secretion of FGF21 might contribute to the observed lower bone mass phenotype in LONP1 mKO mice.

To ascertain the impact of FGF21 on the observed osteogenic inhibition or pro-osteoclastogenic activity in LONP1 mKO serum, we conducted experiments utilizing an FGF21-blocking antibody to neutralize its effects. We observed that serum from LONP1 mKO mice inhibited osteoblast differentiation, but this effect was mitigated when treated with the anti-FGF21-blocking antibody (Fig. 6D and E). Conversely, the FGF21-blocking antibody was also effective in attenuating the pro-osteoclastogenic effects observed in serum from LONP1 mKO mice (Fig. 6F and G). These findings collectively underscore the importance of FGF21 signaling in this regulatory mechanism.

### Serum FGF21 is linked to bone health in humans

**Fig. 7.**
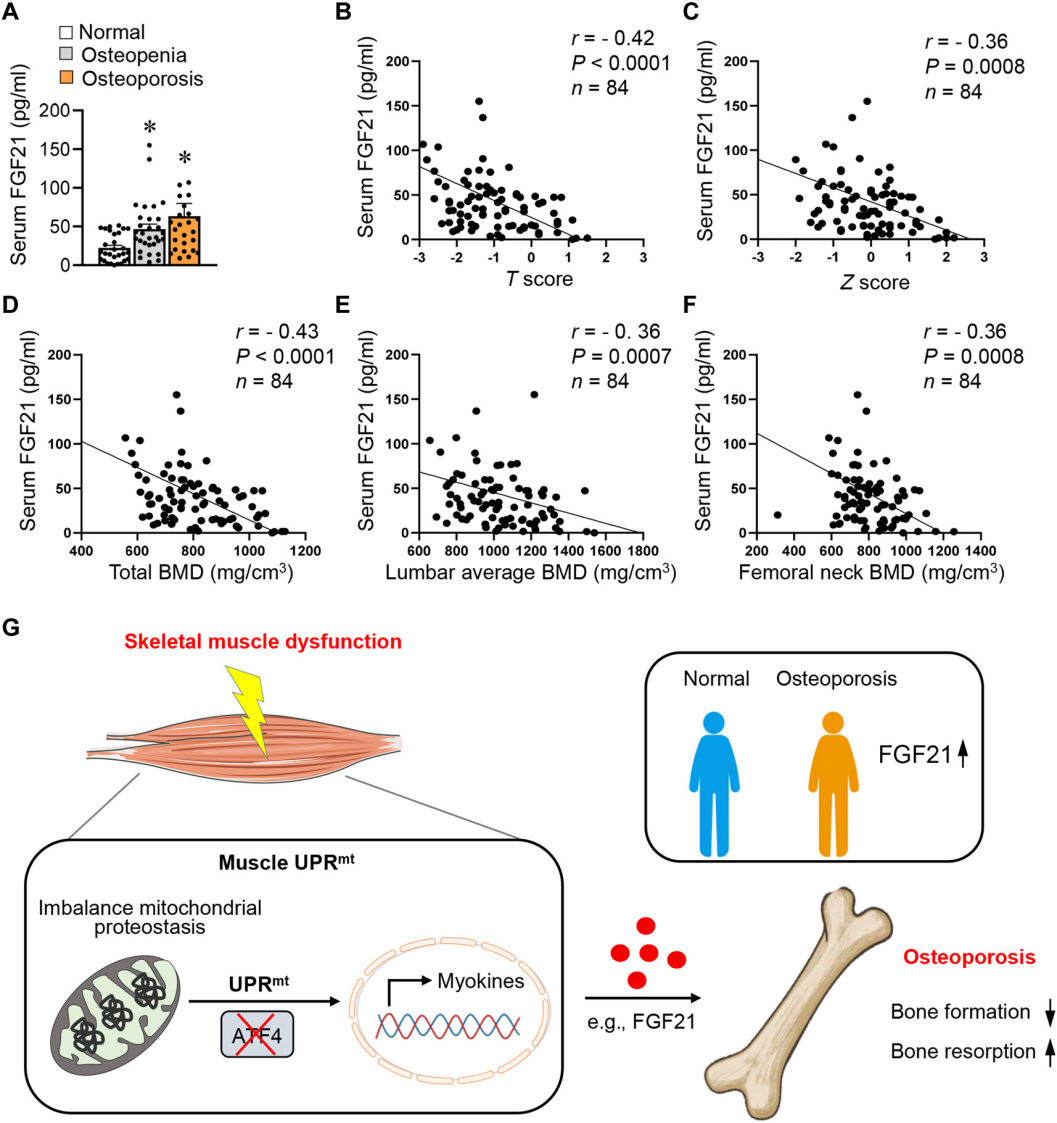
Serum FGF21 is linked to bone health in humans. (A) Serum FGF21 levels detected by ELISA kit (Mlbio, ml058174) in normal, osteopenia, and osteoporosis persons (*n* = 25 to 30). (B to F) Correlations between serum FGF21 levels and *T* score (B), *Z* score (C), total BMD (D), lumbar average BMD (E), or femoral neck BMD (F) (*n* = 84). (G) Model for the LONP1-dependent mitochondrial proteostasis in facilitating muscle–bone crosstalk. Data are shown as the mean ± SEM. *P < 0.05 versus corresponding controls. *P* values were determined by Kruskal–Wallis test with Dunn’s corrections test (A). Spearman’s correlation (B to F) analysis was used to determine the correlation.

To assess the clinical correlation of FGF21 regulatory pathway in humans, we performed an investigation on the relationship between serum FGF21 and bone-related parameters in 84 subjects from the Department of Spine Surgery of Nanjing Drum Tower Hospital. The prevalence of osteoporosis in women aged 50 and older is much higher than in men [42]. As expected, there are markedly more female osteoporosis patients than males (Table S2). Our analysis revealed compelling findings: Individuals with osteoporosis exhibited markedly elevated levels of circulating FGF21 compared to non-osteoporosis subjects (Fig. 7A). To delve deeper into the connection between serum FGF21 levels and bone health, we utilized DXA (dual-energy x-ray absorptiometry), the gold standard for diagnosing osteoporosis, to evaluate various bone-related parameters in these subjects. Remarkably, a negative association was observed between serum FGF21 levels and *T* score (which compares bone density to the average density of healthy 30-year-old adults) and *Z* score (which compares bone density to the average density of people of the same age and gender) (Fig. 7B and C). Additionally, we discovered marked negative associations between serum FGF21 levels and whole-body BMD, lumbar average (L2 to L4) BMD, and femoral neck BMD (Fig. 7D to F). Next, we measured *LONP1* mRNA levels in the muscles. Individuals with osteoporosis exhibited markedly lower muscle *LONP1* levels compared to non-osteoporotic subjects (Fig. S8A). We observed a positive correlation between muscle *LONP1* levels and both the *T* score and *Z* score in humans (Fig. S8B and C). Muscle *LONP1* levels were also positively related to femoral total BMD and neck BMD (Fig. S8D and E).

## Discussion

Skeletal muscle mitochondrial proteostasis stress has the intriguing ability to communicate signals to other tissues, but its potential impact on bone metabolism has remained unclear. In this study, we uncovered that microgravity induces the accumulation of mitochondrial proteins in skeletal muscle, accompanied by reduced levels of LONP1 protein. Our investigation highlights the vital role of LONP1, a mitochondrial protease, in regulating muscle mitochondrial proteostasis, which turns out to be crucial for maintaining bone homeostasis (Fig. 7G). Through skeletal muscle-specific LONP1 deletion, we observed severe bone loss, characterized by heightened bone resorption and diminished bone formation. Additionally, overexpression of the mitochondrial-retained ΔOTC protein in skeletal muscle alone is adequate to trigger bone loss. Our investigations have uncovered a robust connection between mitochondrial proteostasis imbalance and the activation of skeletal muscle UPR^mt^, ultimately leading to bone loss, surprisingly, independent of ATF4. Notably, this bone loss can be attributed to the expression of secreted factors, including FGF21, emanating from the skeletal muscle. Through experiments employing an FGF21-blocking antibody, we further delineated the pivotal role of FGF21 in mediating the regulatory impact of skeletal muscle mitochondrial proteostasis on bone metabolism. These findings establish a direct connection between muscle mitochondrial protein quality control and underscore the essential role of LONP1-dependent mitochondrial proteostasis in facilitating muscle–bone crosstalk.

The connection between muscles and bones goes beyond their mechanical roles, where bones serve as levers and muscles as pulleys, enabling organismal movement. Bones have the capacity to adjust their mass and structure in reaction to mechanical loads imposed by muscles, meaning that degeneration in muscle function can lead to reduced bone density, ultimately causing bone loss [43]. While muscle mass, quantified as lean body mass, contributes markedly to BMD variation, adhering to Wolff’s law, it remains insufficient to account for the entirety of bone mass reduction [44]. Biochemical factors originating from muscles, including myokines [45], are evidently involved in regulating bone mass. In aging individuals, osteoporosis often coincides with sarcopenia or cachexia, yet the precise relationship between these conditions remains unclear [46].

In mammalian cells, mitochondrial proteases are considered the primary mechanism of mitochondrial quality control. Mutations in these proteases have been linked to human genetic diseases [12,14,16,47], emphasizing the importance of regulated mitochondrial protein degradation. Among these proteases, LONP1 mutations have been associated with CODAS syndrome (cerebral, ocular, dental, auricular, skeletal syndrome), a disorder characterized by multisystem malfunctions. The clinical manifestations of CODAS share similarities with other mitochondrial disorders, including ptosis, motor delay, hypotonia, and sensorineural hearing loss. However, CODAS also exhibits distinctive features like skeletal and dental abnormalities [17], prompting speculation that mutations causing loss of function in LONP1 may be implicated in the skeletal anomalies seen in this syndrome. Our study provides compelling evidence that targeted disruption of LONP1 in skeletal muscle results in severe mitochondrial disorders, leading to decreased muscle size and strength and an accelerated aging phenotype typified by osteoporosis. We elucidate the continuous regulatory role of LONP1 in maintaining muscle mass, glucose homeostasis, and bone turnover, supported by findings from inducible skeletal muscle-specific LONP1 knockout mice. Furthermore, our study demonstrates that overexpression of the mitochondrial-retained protein ΔOTC in skeletal muscle activates UPR^mt^ and induces bone loss. These findings suggest that activated UPR^mt^ in skeletal muscle may adversely impact bone health in both male and female mice, highlighting the physiological relevance of disrupted muscle mitochondrial proteostasis and osteoporosis, independent of hormone-related effects. The transcription factor ATF4 is known to play an important role in regulating the mitochondrial stress response in mammals, and its activation directly controls mammalian UPR^mt^ gene expression [38,39]. Surprisingly, we found that muscle mitochondrial proteostasis stress-induced bone loss occurs independently of ATF4. Previous studies have also implicated various other transcriptional signals in the regulation of the mammalian UPR^mt^, such as ATF5 and DDIT3 [38,48,49]. Future research will be necessary to further elucidate the signaling pathways involved in muscle-UPR^mt^.

FGF21, belonging to of the fibroblast growth factor (FGF) family and initially discovered in the liver [50], shares approximately 75% amino acid sequence similarity between mice and humans, demonstrating potential therapeutic benefits in addressing metabolic disorders in preclinical studies. While it holds promise as a therapeutic target for metabolic disorders, recent findings have also reported its potential side effects on bone homeostasis in rodents [51]. Specifically, increased FGF21 expression, via both genetic and pharmacological approaches, has been linked to bone loss and osteoclastogenesis [40,41]. Human studies with FGF21 analogs have produced mixed results regarding bone turnover, with PF-05231023 increasing bone turnover markers, while AKR-001 and pegbelfermin have not [52,53]. In our study, we uncover a marked finding that elevated FGF21 secretion, triggered by skeletal muscle UPR^mt^, induces bone loss independently of ATF4. In addition to the activation of UPR^mt^, the increased expression of FGF21 resulting from LONP1 deletion in muscles may also be due to other factors, such as heightened reactive oxygen levels or metabolic imbalances. Our investigations of serum samples from human osteoporosis patients demonstrated that FGF21 levels were elevated in the osteoporosis group. We also found that serum FGF21 levels were negatively correlated with bone mass measurements. Additionally, muscle LONP1 expression was reduced in osteoporotic individuals and positively correlated with bone parameters, suggesting that the LONP1–FGF21 axis may play a role in bone health in humans. Another markedly induced mitokine in LONP1 mKO and MCK-ΔOTC muscles is GDF15, which is known for its favorable effects on systemic energy metabolism [54–56]. Indeed, we also observed markedly higher *Gdf15* levels in LONP1 mKO and MCK-ΔOTC muscles compared to the controls. Whether muscle-derived GDF15 affects bone metabolism requires further investigation in the future.

In general, our finding illuminates the vital role of LONP1-mediated mitochondrial proteostasis in preserving bone metabolic equilibrium, offering potential insights into the intricate interplay between mitochondria and bone health and promising therapeutic avenues for osteoporosis treatment.

## Methods

### Mouse models

All animal studies were conducted in strict accordance with the institutional guidelines for the humane treatment of animals and were approved by the Institutional Animal Care and Use Committee at the Model Animal Research Center of Nanjing University (approval no. GZJ11). Muscles (GC, WV, TA, Sol), bone, adipose tissues, liver, heart, and blood were collected from the mice. Male C57BL/6J WT mice were procured from GemPharmatech. Lonp1^f/f^ mice, LONP1 mKO mice, MCK-△OTC mice, and LONP1/ ATF4 DmKO mice were generated as previously described [57]. MCK-△OTC mice were compared to NTG littermate controls. To achieve tamoxifen-inducible muscle-specific deletion of LONP1 (LONP1^HSA-MCM^ ) mice [58], LONP1^f/f^ mice were bred with mice carrying a chimeric Cre recombinase that incorporates a mutated estrogen receptor ligand-binding domain, with the drive of HSA promoter. Offspring underwent genotyping, and mice aged from 8 to 18 weeks were utilized. The mice were provided with unlimited access to either standard laboratory rodent CD or an HFD comprising 60% of calories from fat (Research Diets no. D12492) for 5 weeks (HFD-fed WT and HFD-fed LONP1 mKO mice) or 20 weeks (HFD-fed LONP1^f/f^ and HFD-fed LONP1^HSA-MCM^ mice).

### Human studies

In this study, 84 patients aged over 50 undergoing spinal operations in our department between September 2021 and September 2022 were enrolled. All patients were assessed via DXA evaluation for osteoporosis. Those with a history of severe infectious or systemic autoimmune diseases were not included from the study. The research received approval from the ethical committee of Nanjing Drum Tower Hospital (institutional review board no. 2021-398-01). Deep paraspinal muscle biopsies were taken during spinal surgery, cleaned, and flash frozen in liquid nitrogen for RNA isolation. Preoperative fasting morning blood samples were extracted and centrifuged (3,000 rpm, 5 min). The plasma samples were obtained by carefully aspirating the supernatant and stored at −80 °C until use.

### Primary cell culture

Primary osteoclasts and osteoblasts were prepared from mice following a previously published protocol [59]. Briefly, femur and tibia bones were obtained, cleansed to remove muscle and soft tissue, and then transferred to the culture medium. The bone tissue was incised from both ends, and BMCs were harvested by rinsing with a 1-ml syringe, which were then inoculated in culture medium [α-MEM (minimum essential medium) containing penicillin, streptomycin, and 10% fetal bovine serum]. After 16 to 24 h, cell suspension was centrifuged at 1,000 rpm for 5 min and resuspended in α-MEM culture medium supplied by 10 ng/ml M-CSF (PeproTech) for 2 d to gain BMDMs. The adherent cells (BM-MSCs) were maintained in α-MEM culture medium for future osteogenic differentiation. For osteoclast differentiation, BMDMs were reseeded in a 96-well dish (2 × 10^4^ cells per well) and cultured with α-MEM medium supplied by 10 ng/ml M-CSF combined with 50 ng/ml RANKL (R&D Systems) for 5 d, with medium replacement every 2 d. Osteoclasts were fixed in 4% paraformaldehyde (PFA) by 10 min, then washed by phosphate-buffered saline (PBS), and stained with TRAP satin solution (Wako) at 37 °C for 30 min. Osteoclasts were characterized as TRAP^+^ cells (≥3 nuclei) [41]. For osteoblast differentiation, BM-MSCs were seeded in 12-well plates and cultured in osteogenic medium (100 mM ascorbic acid, 5 mM β-glycerophosphate, and 10 nM dexamethasone) for 7 d, with medium replacement every 3 d. ALP staining was performed for osteoblast activity measurement. Briefly, osteoblasts were fixed in 4% PFA for a duration of 10 min, then washed by PBS, and stained with ALP staining solution (Wako) to assess osteoblast activity.

### Micro-CT analysis

Micro-CT analysis was conducted as previously outlined [60,61]. Briefly, male LONP1 mKO, LONP1^HSA-MCM^, MCK-ΔOTC, LONP1/ATF4 DmKO mice and female LONP1 mKO, MCK-ΔOTC mice were sacrificed. The femurs and fifth LVs were cleaned of soft tissues, fixed overnight in 4% PFA, and rinsed with PBS prior to scanning. Micro-CT analysis was performed on femoral trabecular bones to assess the BV and 3D bone architecture using a Scanco scanner (Viva80 microCT, SCANCO Medical) following the manufacturer’s instructions. The scanner settings were configured with a current of 56 μA, a voltage of 70 kVp, and a voxel size of 15.6 μm. For 3D histomorphometric analysis utilizing 3D distance techniques (Scanco Medical), 80 slices of trabecular bone underneath the growth plate of the distal femur and 40 slices of trabecular bone underneath the superior cortical endplate of the fifth LV were chosen.

### Biomechanical analysis

Three-point bending strength test was conducted as previously outlined [62]. Bones from male LONP1 mKO, LONP1^HSA-MCM^, MCK-ΔOTC, and LONP1/ATF4 DmKO mice were tested using a biomechanical machine (INSTRON E3000, Instron, USA). In brief, femurs were positioned on 2 fulcrums spaced 10 mm apart, and a load stress of 0.5 N was evenly applied to the femur’s midpoint. A loading bar gradually exerted force at a constant rate of 0.02 mm/s until the femur specimen fractured. Failure occurred directly beneath the loading point, at the midpoint of the femur. The data were analyzed using the software provided by the mechanical experiment tester. Measured parameters include ultimate force [maximal load, measured in newtons (N)], compressive ultimate stress (the stress required to rupture a specimen, measured in MPa), post-yield displacement (the displacement from the yield point to the fracture point, measured in mm), and elastic modulus (Young’s modulus, measured in MPa).

### Calcein labeling

Calcein labeling was conducted following previously outlined procedures [63,64]. Intraperitoneal calcein injection (25 mg/kg; Sigma) was used to assess dynamic bone formation, 10 and 2 d prior to harvesting. Bones from male LONP1 mKO and MCK-ΔOTC mice were embedded in OCT (optimal cutting temperature compound), frozen at −80 °C, and cryosectioned using a Leica CM 3050S cryostat with installed CryoJane. Frozen tissue sections (10 μm) were moved onto ^1^/_2_ type commercial CryoJane slides (glass-CJ slides). The tape-section combination was swiftly transferred into the chilly stream at the rear of the cryostat and placed onto a coated glass slide on the curing platform. Then, an ultraviolet pulse was administered for a duration of 8 to 10 s. The histology slides were analyzed and photographed using a fluorescent microscope, and MAR was calculated by dividing the distance between 2 calcein labels by the time interval between the 2 injections.

### Histological analysis

Femurs from male LONP1 mKO, LONP1^HSA-MCM^, MCK-ΔOTC, and LONP1/ATF4 DmKO mice were fixed overnight in 4% PFA at 4 °C. Subsequently, samples were then decalcified in 15% EDTA for 14 d and embedded in paraffin as previously described [34,65]. Paraffin slices with a thickness of 8 μm were subjected to H&E staining for histology analysis. For TRAP and ALP staining, the slices were incubated in the working solution (Wako) for 0.5 to 2 h at room temperature. Isopentane cooled in liquid nitrogen was used to freeze muscle tissue, which was subsequently stained with fluorescein isothiocyanate-conjugated wheat germ agglutinin (WGA; Sigma-Aldrich, #L4859). Livers of the mice were embedded in OCT, frozen at −80 °C, and then subjected to 10-μm-thick slices. The slices were subsequently stained with 0.5% Oil Red O and counterstained with H&E.

### Tamoxifen treatment

Tamoxifen treatment was performed as previously described [66].The male LONP1^HSA-MCM^ and LONP1^f/f^ littermate controls received intraperitoneal injections of tamoxifen (Sigma, USA) diluted in corn oil (15 mg/ml) in a dose of 100 mg/kg with 7-day injection. The LONP1^f/f^ and LONP1^HSA-MCM^ male mice fed with a standard CD were injected by tamoxifen at 6 weeks old and harvested at 15 weeks old. The HFD-fed LONP1^f/f^ and LONP1^HSA-MCM^ male mice were fed HFD 20 weeks from 6 weeks old, treated with tamoxifen at 17 weeks old (noticeable increase in weight), and harvested at 26 weeks old.

### Glucose and insulin tolerance testing

The male LONP1^HSA-MCM^ and LONP1^f/f^ mice underwent an overnight fast for glucose tolerance tests (GTTs) or a 4-h fast for insulin tolerance tests (ITTs). For GTT analysis, 1.5 g/kg of d-glucose was administered via intraperitoneal injection. For ITT, 0.75 U/kg of insulin (Sigma-Aldrich) was administered through intraperitoneal injection. Levels of blood glucose were assessed at 0, 15, 30, 60, 90, and 120 min after challenge with a OneTouch UltraMini glucose meter (OneTouch).

### RNA analyses

Quantitative RT-PCR (qRT-PCR) was conducted as previously described [34,36]. Briefly, muscle tissues from male LONP1 mKO, MCK-ΔOTC, and LONP1/ATF4 DmKO mice or humans were homogenized and processed for total RNA extraction using RNAiso Plus (9109, Takara Bio). The purified RNA samples were then reverse transcribed using the PrimeScript RT Reagent Kit with gDNA Eraser (RR047, Takara Bio). Following reverse transcription, qRT-PCR was carried out using the ABI Prism Step-One system with the SYBR Green Master-Mix (RR420, Takara Bio). The oligonucleotide primers specific to the target gene sequences were provided in Table S2. Arbitrary units of target mRNA were corrected to the expression of 36b4.

### ELISA

Serum osteocalcin (Novus,NBP2-68151), CTX-1 (Cloud-Clone, CEA665Mu), and FGF21 (Proteintech, KE10042) in male LONP1 mKO, LONP1^HSA-MCM^, MCK-ΔOTC, and LONP1/ATF4 DmKO mice and serum FGF21 (Mlbio, ml058174) in humans were conducted using enzyme-linked immunosorbent assay (ELISA) kits. Serum samples were diluted at 1:5 (Osteocalcin), 1:2.5 (CTX-1), or 1:4 (FGF21) for mice and 1:1 for humans, following the manufacturer’s instructions.

### Antibodies and immunoblotting studies

Antibodies used were β-actin (4970, 1:1,000; CST), LONP1 (15440-1-AP, 1:2,500; Proteintech), and Peroxidase AffiniPure Goat Anti-Rabbit IgG (H+L) (Jackson 111-0350003). The FGF21 neutralizing antibody (Antibody and Immunoassay Services, HKU) was used in some experiments. Western blotting studies were performed as previously described [67]. Briefly, tissues were homogenized with homogenizer (Jingxin, Shanghai) in ice-cold radioimmunoprecipitation assay (RIPA) buffer [150 mM NaCl, 20 mM tris–HCl (pH 7.4), 1 mM EDTA, 1 mM sodium, 100 mM phenylmethylsulfonyl fluoride, 500 mM NaF, 200 mM Na_3_VO_4_, 10× complete]. Total protein concentration was assessed with Pierce BCA Assay Kit Protocol (Thermo Fisher Scientific). Equal total protein (20 μg) was loaded to each lane. Blots were normalized to β-actin.

### Quantification and statistical analysis

Statistical analyses in mouse and cell studies were performed using GraphPad Prism 8.0.1 software. All mouse and cell studies were analyzed by 2-tailed Student’s *t* test when comparing 2 groups or analysis of variance (ANOVA) coupled to a Fisher’s least significant difference (LSD) post hoc test when more than 2 groups were compared. For GO pathway analysis, the filtered datasets were uploaded into DAVID Bioinformatics Resources 6.9 and Fisher’s exact test is adopted to measure the *P* values of gene enrichment in annotation terms. Sample sizes (ranging from *n* = 3 to 8) were included in the figures. Statistical analyses in human studies were performed using GraphPad Prism 8.0.1 software. The Kolmogorov–Smirnov test examined the normality of continuous variables. For data that followed a normal distribution, 2-tailed Student’s *t* test was used for 2-group comparisons. For data that did not follow a normal distribution, Kruskal–Wallis test with Dunn’s corrections test was used for comparisons among multiple groups. Correlations were assessed using Pearson’s correlation test or Spearman’s correlation test. All data points were utilized in statistical analyses and are depicted as mean ± SEM, with statistical significance set as *P* < 0.05.

## Data Availability

The data are available from the authors upon a reasonable request.
