# Supplementary material for: Imbalanced Skeletal Muscle Mitochondrial Proteostasis Causes Bone Loss
Source: Research (Wash D C). 2024 Aug 30;7:0465. doi: 10.34133/research.0465 (PMC11362843; doi:10.34133/research.0465)
Supplement: Supplementary 1 — Figs. S1 to S8 Tables S1 to S3 [file research.0465.f1.docx]

**
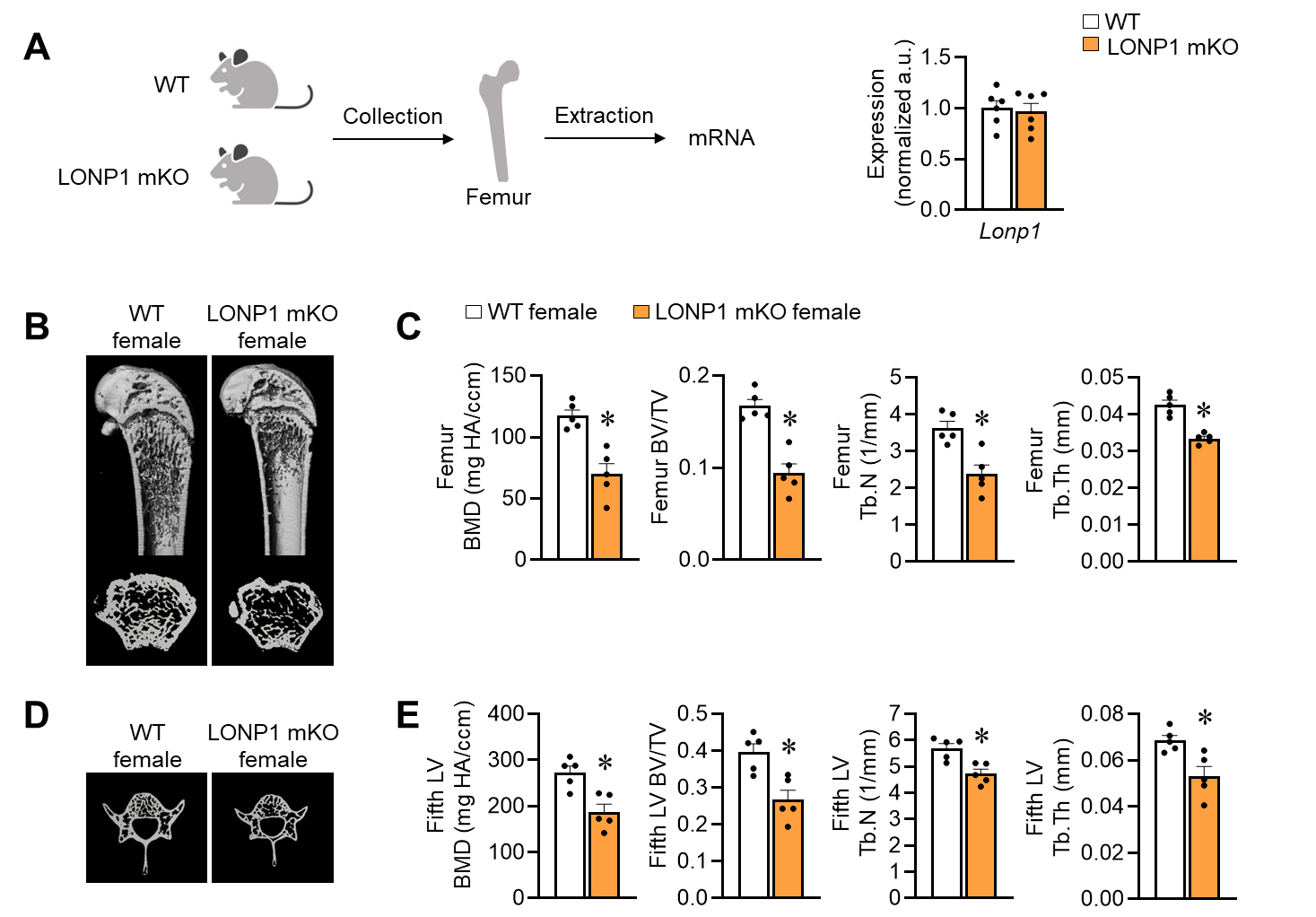
Fig. S1 Skeletal muscle-specific deletion of LONP1 caused bone loss in female mice, related to Fig. 1.**

**(A)** Schematic representation for extraction of femur mRNA and relative mRNA expression of Lonp1 from WT and LONP1 mKO male mice (n = 8).

**(B-E)** WT and LONP1 mKO female mice were harvested at 8 weeks old.

**(B)** Representative µCT images of the trabecular bone in the distal femur metaphysis (n = 5).

**(C)** Quantitative analysis of BMD, BV/TV, Tb.N and Tb.Th in femurs (n = 5).

**(D)** Representative µCT images of the trabecular bone in the fifth LVs (n = 5).

**(E)** Quantitative analysis of BMD, BV/TV, Tb.N and Tb.Th in the fifth LVs (n = 5).

Data are shown as the mean ± SEM. **P* < 0.05 vs. corresponding controls. *P* values were determined by an unpaired two-tailed Student’s t-test.

**
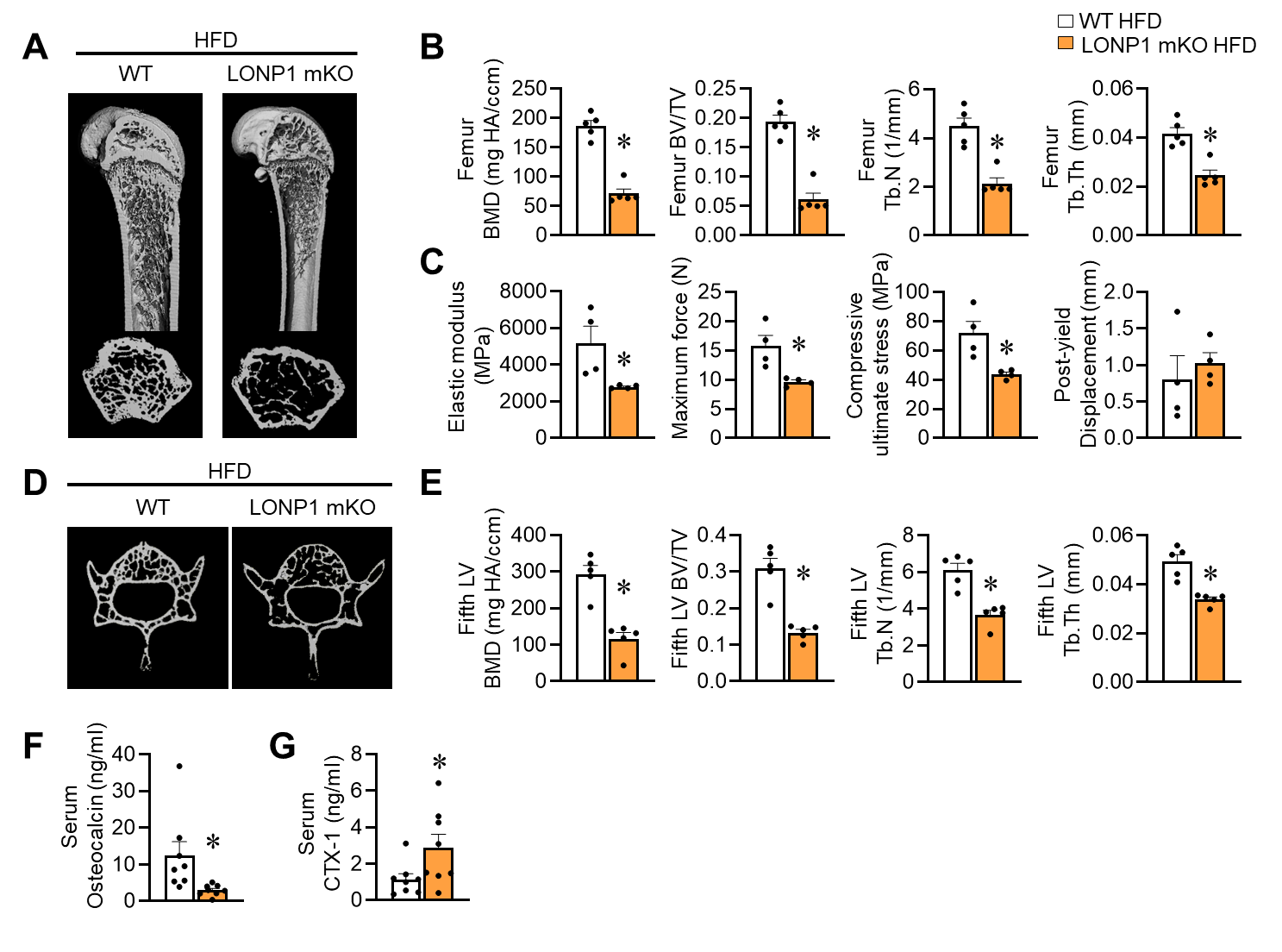
**

**Fig. S2 Skeletal muscle-specific deletion of LONP1 caused bone loss during HFD, related to Fig. 1.**

**(A-G)** WT and LONP1 mKO male mice were fed HFD 5 weeks and harvested at 11 weeks old.

**(A)** Representative µCT images of the trabecular bone in the distal femur metaphysis from HFD-fed WT and HFD-fed LONP1 mKO mice.

**(B)** Quantitative analysis of BMD, BV/TV, Tb.N and Tb.Th in femurs from HFD-WT and HFD-LONP1 mKO mice (n = 5).

**(C)** Bending tests analysis of elastic modulus, maximum force, compressive ultimate stress and post-yield displacement of femurs from HFD-fed WT and HFD-fed LONP1 mKO mice (n = 4).

**(D)** Representative µCT images of the trabecular bone in the fifth LVs from HFD-fed WT and HFD-fed LONP1 mKO mice.

**(E)** Quantitative analysis of BMD, BV/TV, Tb.N and Tb.Th in the fifth LVs from HFD-fed WT and HFD-fed LONP1 mKO mice (n = 5).

**(F)** Serum P1NP levels in HFD-fed WT and HFD-fed LONP1 mKO mice (n = 8).

**(G)** Serum CTX-1 levels in HFD-fed WT and HFD-fed LONP1 mKO mice (n = 8).

Data are shown as the mean ± SEM. **P* < 0.05 vs. corresponding controls. *P* values were determined by an unpaired two-tailed Student’s t-test.

**
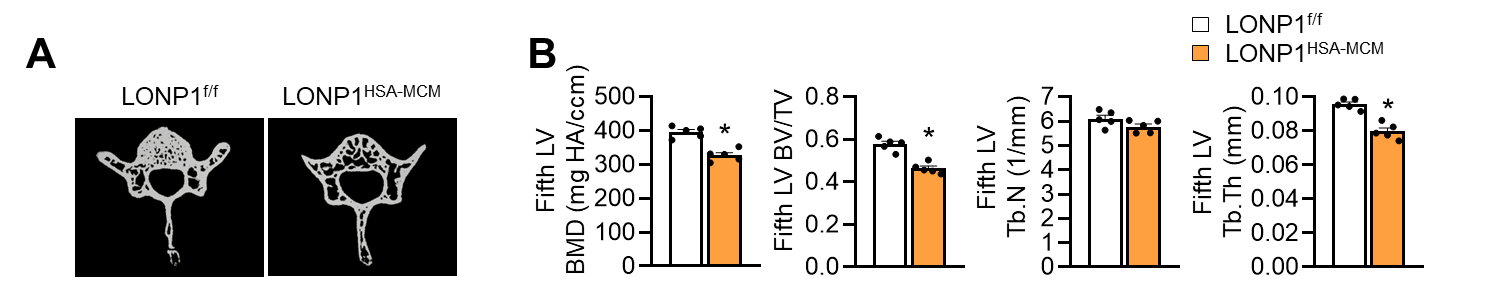
**

**Fig. S3 The sudden loss of LONP1 in mature muscles** **leads to bone loss in the fifth LVs, related to Fig. 2.**

**(A and B)** LONP1^f/f^ and LONP1^HSA-MCM^ male mice were treated with tamoxifen at 6 weeks old and harvested at 15 weeks old.

**(A)** Representative µCT images of the trabecular bone in fifth LVs from LONP1^f/f^ and LONP1^HSA-MCM^ mice.

**(B)** Quantitative analysis of BMD, BV/TV, Tb.N and Tb.Th in the fifth LVs from LONP1^f/f^ and LONP1^HSA-MCM^ mice (n = 5 mice per group).

Data are shown as the mean ± SEM. **P* < 0.05 vs. corresponding controls. *P* values were determined by an unpaired two-tailed Student’s t-test.

**
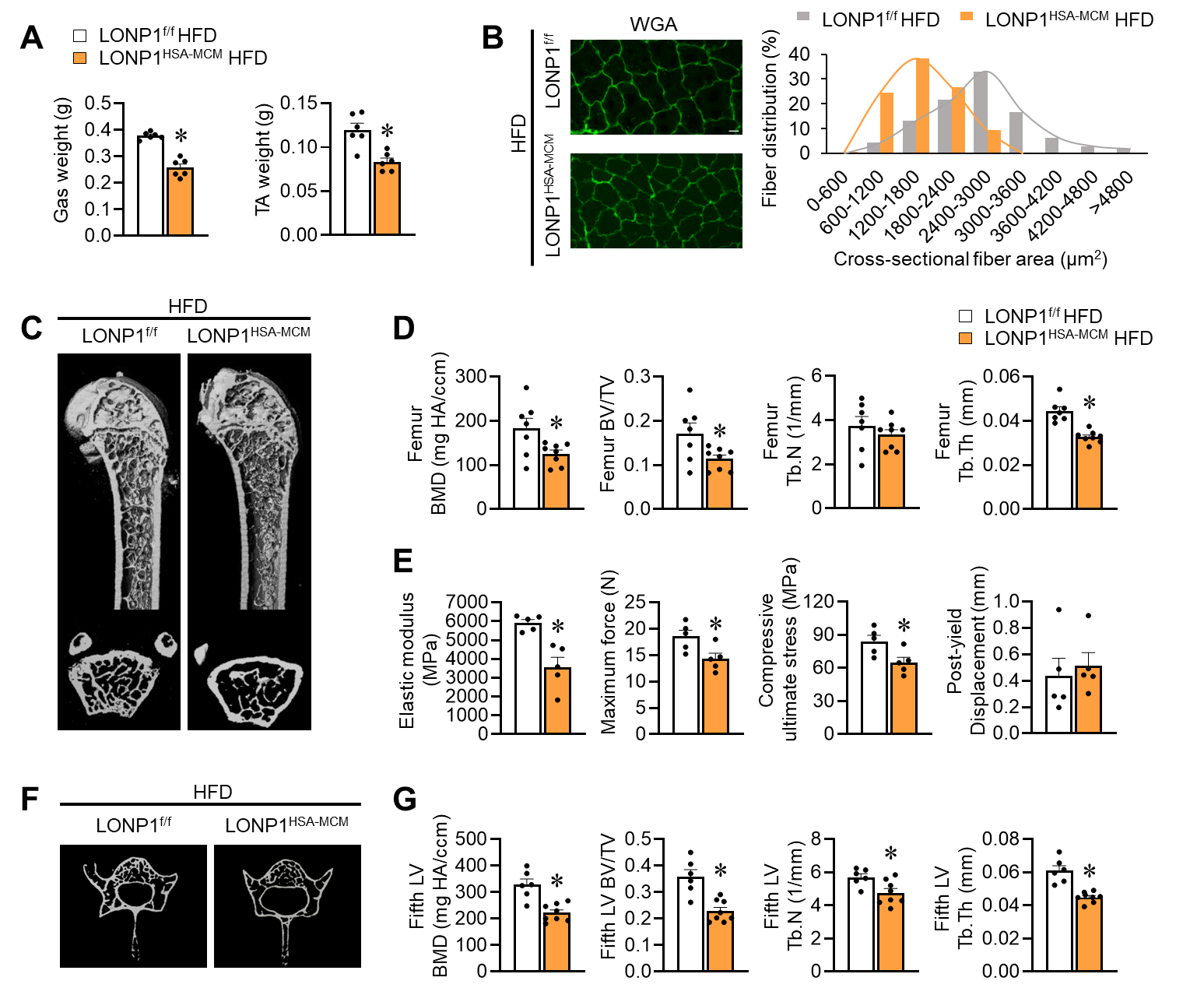
**

**Fig. S4 The sudden loss of LONP1 caused bone loss during HFD, related to Fig. 1.**

**(A-G)** LONP1^f/f^ and LONP1^HSA-MCM^ male mice were fed HFD 20 weeks, treated with tamoxifen at 17 weeks old and harvested at 26 weeks old.

**(A)** GC and TA muscles weight (n = 6).

**(B)** The staining of GC muscle with WGA (scale bar, 20μm) and quantification of fiber size distribution (n = 5-6).

**(C)** Representative µCT images of the trabecular bone in the distal femur metaphysis from HFD-fed LONP1^f/f^ and HFD-fed LONP1^HSA-MCM^ mice.

**(D)** Quantitative analysis of BMD, BV/TV, Tb.N and Tb.Th in femurs from HFD-fed LONP1^f/f^ and HFD-fed LONP1^HSA-MCM^ mice (n = 7-8 mice per group).

**(E)** Bending tests analysis of elastic modulus, maximum force, compressive ultimate stress and post-yield displacement of femurs from HFD-fed LONP1^f/f^ and HFD-fed LONP1^HSA-MCM^ mice (n = 5 mice per group).

**(F)** Representative µCT images of the trabecular bone in the fifth LVs from HFD-fed LONP1^f/f^ and HFD-fed LONP1^HSA-MCM^ mice.

**(G)** Quantitative analysis of BMD, BV/TV, Tb.N and Tb.Th in the fifth LVs from HFD-fed LONP1^f/f^ and HFD-fed LONP1^HSA-MCM^ mice (n = 6-8 mice per group).

Data are shown as the mean ± SEM. **P* < 0.05 vs. corresponding controls. *P* values were determined by an unpaired two-tailed Student’s t-test.

**
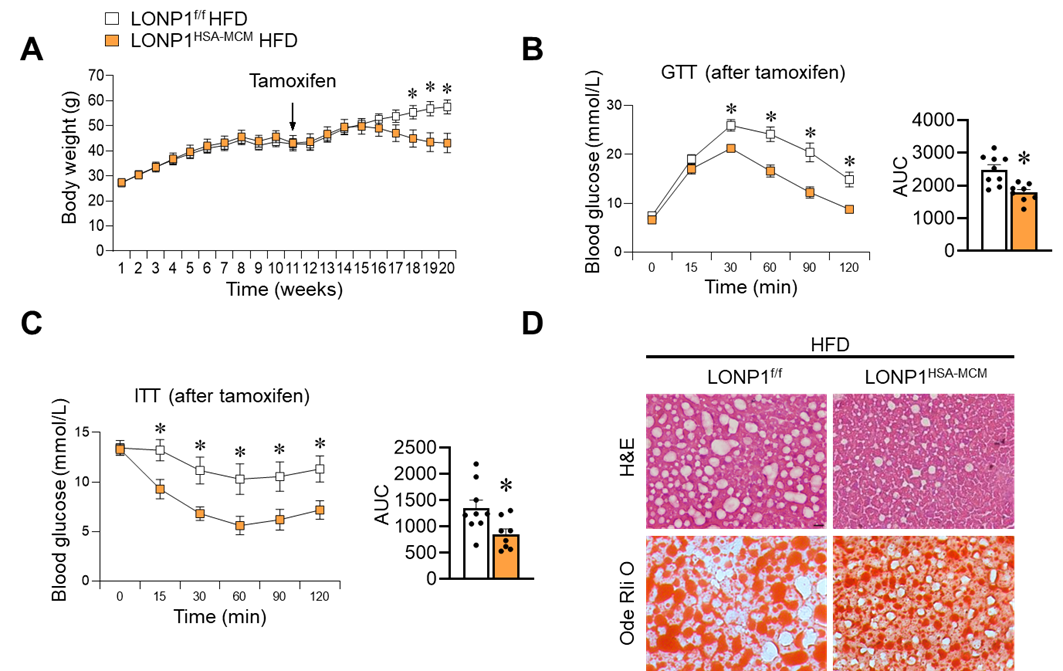
**

**Fig. S5 Acute LONP1 deletion in skeletal muscle impacts diet-induced obesity and insulin resistance**

**(A-D)** LONP1^f/f^ and LONP1^HSA-MCM^ male mice were fed HFD 20 weeks, treated with tamoxifen at 17 weeks old and harvested at 26 weeks old.

**(A)** Body weight following HFD feeding and tamoxifen treatment (n = 8-9 mice per group).

**(B)** Left: Glucose tolerance test (GTT and ITT). Right: The area under the curve for GTT is shown (n = 8-9 mice per group).

**(C)** Left: Insulin tolerance test (ITT). Right: The area under the curve for ITT is shown (n = 8-9 mice per group).

**(D)** H&E and Oil Red O staining of livers (scale bar, 20μm, n = 5-6 mice per group).

Data are shown as the mean ± SEM. **P* < 0.05 vs. corresponding controls. *P* values were determined by an unpaired two-tailed Student’s t-test (**B Right** and **C Right**) or by two-way ANOVA (**A**, **B Left** and **C Left**).

**
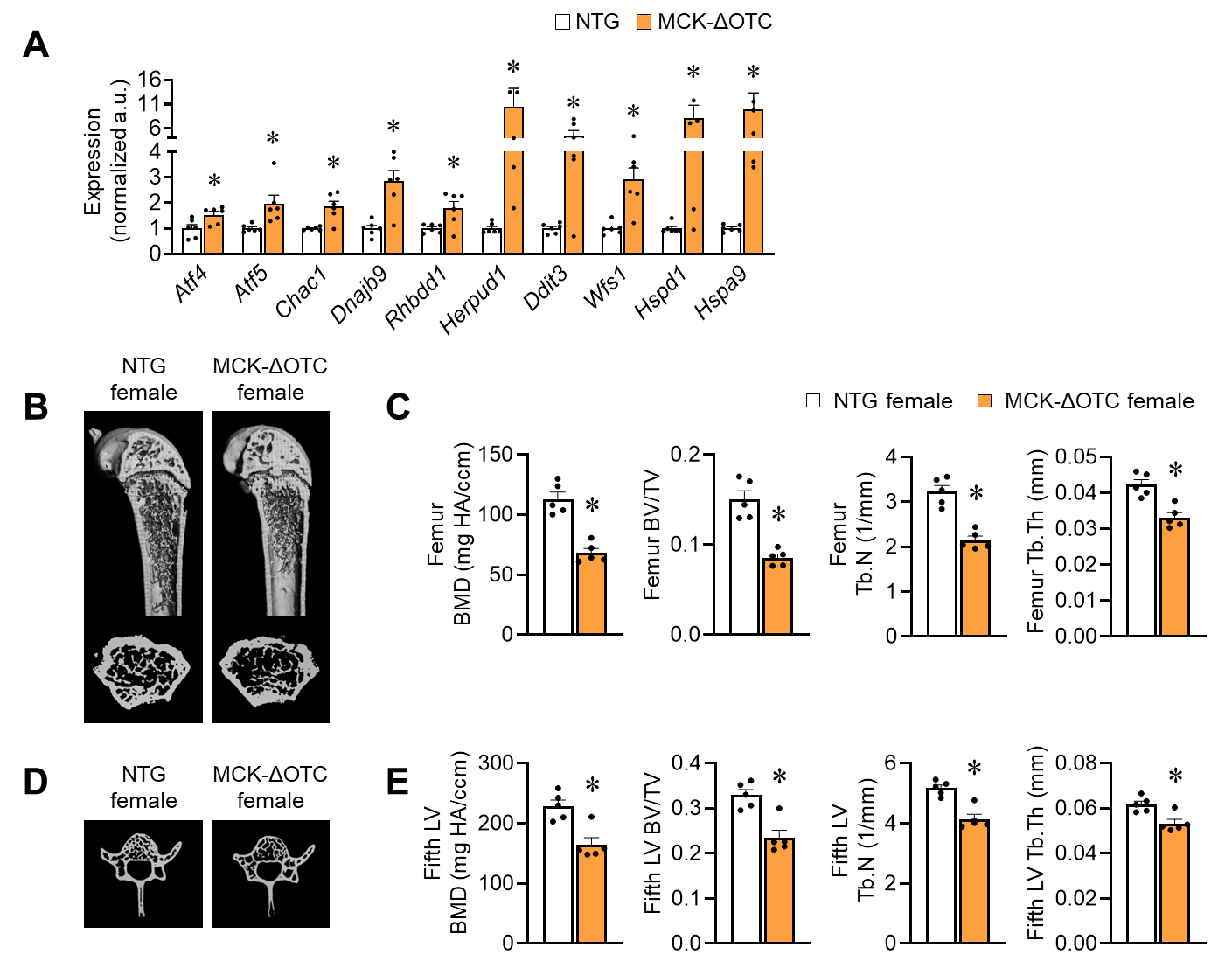
Fig. S6 Skeletal muscle-specific overexpression of mitochondrial-retained ΔOTC protein induces bone loss in female mice, related to Fig. 3.**

**(A)** Expression of genes related to UPR^mt^ in GC muscles from NTG and MCK-ΔOTC mice (n = 6).

**(B-E)** NTG and MCK-ΔOTC female mice were harvested at 8 weeks old.

**(B)** Representative µCT images of the trabecular bone in the distal femur metaphysis.

**(C)** Quantitative analysis of BMD, BV/TV, Tb.N and Tb.Th in femurs (n = 5 mice per group).

**(D)** Representative µCT images of the trabecular bone in the fifth LVs.

**(E)** Quantitative analysis of BMD, BV/TV, Tb.N and Tb.Th in the fifth LVs (n = 5 mice per group).

Data are shown as the mean ± SEM. **P* < 0.05 vs. corresponding controls. *P* values were determined by an unpaired two-tailed Student’s t-test.

**
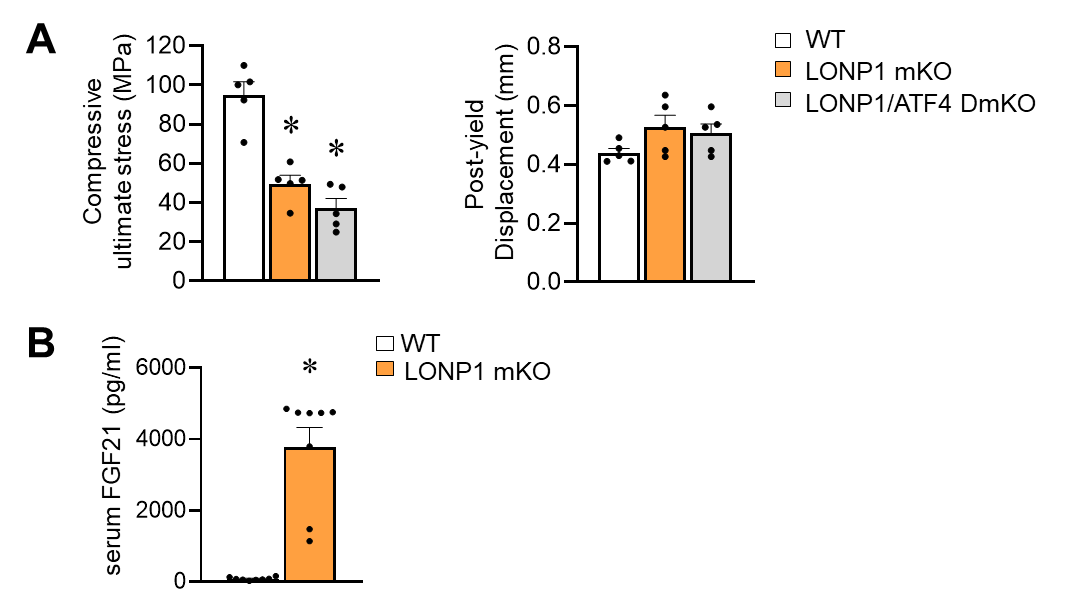
**

**Fig. S7 Mitochondrial proteostasis stress in muscle induced bone loss independent of ATF4 and FGF21 is associated with bone loss in muscle LONP1-deficient mice, related to Fig. 5 and 6.**

**(A)** Bending tests analysis of compressive ultimate stress and post-yield displacement of in femurs from WT, LONP1 mKO and LONP1/ATF4 DmKO mice (n = 5).

**(B)** Serum FGF21 level in WT and LONP1 mKO mice (n = 8).

Data are shown as the mean ± SEM. **P* < 0.05 vs. corresponding controls. *P* values were determined by one-way ANOVA (**A**) or by an unpaired two-tailed Student’s t-test (**B**).

**
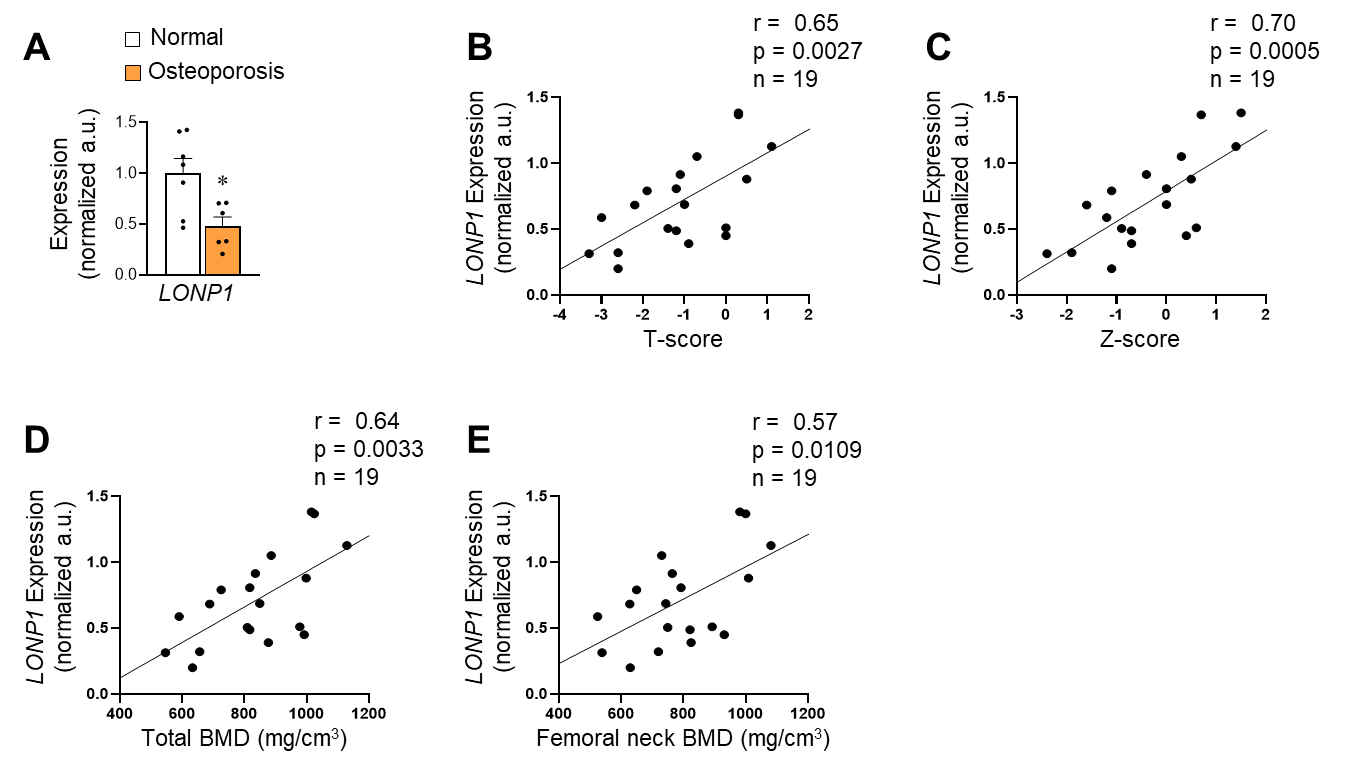
**

**Fig. S8 Skeletal muscle LONP1 is linked to bone health in humans**

**(A)** Relative mRNA expression of skeletal muscle *LONP1* in normal and osteoporosis persons (n = 6-7).

**(B-E)** Correlations between relative mRNA expression of skeletal muscle *LONP1* and T-score (B), Z-score (C), total BMD (D), or femoral neck BMD (E) (n = 20).

Data are shown as the mean ± SEM. **P* < 0.05 vs. corresponding controls. *P* values were determined by an unpaired two-tailed Student’s t-test (A). Pearson’s correlation (B-E) analysis was used to determine the correlation.

**Table S1. Myokines genes.**

| **Gene symbol** | **Description** | **Fold change (vs. Control)** | |
| --- | --- | --- | --- |
|  |  | **LONP1 mKO** | **MCK-ΔOTC** |
| *Gdf15* | *growth differentiation factor 15* | *320.3* | *634.2* |
| *Cdsn* | *corneodesmosin* | *290.9* | *115.7* |
| *Fgf21* | *fibroblast growth factor 21* | *276.7* | *713.8* |
| *S100a8* | *S100 calcium binding protein A8* | *76.1* | *54.3* |
| *Smpdl3b* | *sphingomyelin phosphodiesterase acid like 3B* | *9* | *7* |
| *Lgals3* | *galectin 3* | *7.5* | *40.6* |
| *Igfbp2* | *insulin like growth factor binding protein 2* | *6.2* | *3.5* |
| *Fgf7* | *fibroblast growth factor 7* | *5.8* | *4.6* |
| *Nog* | *noggin* | *5.5* | *14.1* |
| *Ostn* | *osteocrin* | *5.2* | *4.9* |
| *Stc2* | *stanniocalcin 2* | *4.9* | *2.1* |
| *Gdf9* | *growth differentiation factor 9* | *4.8* | *2.7* |
| *Ern1* | *endoplasmic reticulum to nucleus signaling 1* | *3.9* | *4.1* |
| *Masp1* | *mannan binding lectin serine peptidase 1* | *3.8* | *2.5* |
| *Ctsl* | *cathepsin L* | *3.7* | *7.3* |
| *Ero1l* | *endoplasmic reticulum oxidoreductase 1 alpha* | *3.5* | *3.4* |
| *Lgi2* | *leucine rich repeat LGI family member 2* | *3.5* | *2.4* |
| *Hhipl1* | *HHIP like 1* | *3.3* | *3* |
| *Cxadr* | *CXADR Ig-like cell adhesion molecule* | *3.2* | *4.1* |
| *Lcn2* | *lipocalin 2* | *3.1* | *46.2* |
| *Gpnmb* | *glycoprotein nmb* | *2.8* | *6.5* |
| *Rnd1* | *Rho family GTPase 1* | *2.4* | *2* |
| *Extl1* | *exostosin like glycosyltransferase 1* | *2.4* | *3.6* |
| *Angptl6* | *angiopoietin like 6* | *2.3* | *4* |
| *Mamdc2* | *MAM domain containing 2* | *2.2* | *1.9* |
| *Dhrs7* | *dehydrogenase/reductase 7* | *2.1* | *1.8* |
| *Sfn* | *stratifin* | *2.1* | *1.9* |
| *Scpep1* | *serine carboxypeptidase 1* | *2* | *3.4* |
| *Mks1* | *MKS transition zone complex subunit 1* | *2* | *1.5* |
| *Tor3a* | *torsin family 3 member A* | *2* | *1.9* |
| *Gpc4* | *glypican 4* | *1.9* | *1.5* |
| *Irak2* | *interleukin 1 receptor associated kinase 2* | *1.8* | *1.9* |
| *Art5* | *ADP-ribosyltransferase 5* | *1.8* | *1.7* |
| *Cx3cl1* | *C-X3-C motif chemokine ligand 1* | *1.8* | *1.7* |
| *Dnajb9* | *DnaJ heat shock protein family (Hsp40) member B9* | *1.7* | *3* |
| *Bhlhb9* | *basic helix-loop-helix family member b9* | *1.7* | *1.6* |
| *Pam* | *peptidylglycine alpha-amidating monooxygenase* | *1.7* | *1.7* |
| *Litaf* | *lipopolysaccharide induced TNF factor* | *1.7* | *2* |
| *Gfod1* | *glucose-fructose oxidoreductase domain containing 1* | *1.5* | *1.7* |
| *Manf* | *mesencephalic astrocyte derived neurotrophic factor* | *1.5* | *1.8* |

**Table S2. Human subject characteristics**

|  | Normal | Osteopenia | Osteoporosis |
| --- | --- | --- | --- |
| Gender (male/female) | 18/11 | 8/22 | 5/20 |
| Age (yr) | 63.3 ± 1.4 | 65.5 ± 1.3 | 66.8 ± 1.6 |
| CRP (mg/L) | 4.4 ± 1.3 | 7.5 ± 2.4 | 5.2 ± 2.2 |
| ALT (u/L) | 26.4 ± 4.7 | 20.2 ± 2.9 | 19 ± 1.9 |
| AST (u/L) | 24.6 ± 2.4 | 23.2 ± 1.5 | 22.1 ± 1.4 |
| Urea (mmol/L) | 5.9 ± 0.3 | 6.0 ± 0.3 | 5.6 ± 0.3 |
| Creatinine (μmol/L) | 63.2 ± 2.8 | 60.5 ± 2.9 | 55.1 ± 2.4* |
| Total BMD (g/cm2) | 957.9 ± 17.4 | 760.7 ± 11.9* | 679.4 ± 17.5* |
| T-score | 0.2 ± 0.1 | -1.3 ± 0.1* | -1.9 ± 0.1* |
| Z-score | 0.8 ± 0.1 | -0.3 ± 0.1* | -0.8 ± 0.1* |
| Lumbar average BMD (mg/cm3) | 1225.5 ± 26.7 | 1023.3 ± 18.9* | 828.1 ± 20.6* |
| Femoral neck BMD (mg/cm3) | 956.6 ± 19.2 | 771.5 ± 14.3* | 682.1 ± 24.5* |
| serum FGF21 (pg/ml) | 22.4 ± 3.3 | 46.7 ± 6.3* | 63.3 ± 16.5* |

BMD: bone mineral density. CRP: C-reactive protein. ALT: Alanine aminotransferase. AST: Aspartate aminotransferase. IGF2: Insulin growth factor 2. Data are shown as the mean ± SEM. **P* < 0.05 vs. corresponding controls. *P* values were determined by Kruskal-Wallis test with Dunn’s corrections test (Age, CRP, ALT, AST, Urea, Creatinine, serum FGF21) or one-way ANOVA (Total BMD, T-score, Z-score, Lumbar average BMD, Femoral neck BMD).

**Table S3. RT-qPCR primers.**

| **Supplementary Table 3 RT-qPCR primers** | | |
| --- | --- | --- |
| ***Mouse Gene*** | ***Forward*** | ***Reverse*** |
| *36b4* | *5’-ATCCCTGACGCACCGCCGTGA* | *5’-TGCATCTGCTTGGAGCCCACGT* |
| *Lonp1* | *5’-CATTGCCTTGAACCCTCTC* | *5’-ATGTCGCTCAGGTAGATGG* |
| *Fgf21* | *5’-TACACAGATGACGACCAAGA* | *5’-GGCTTCAGACTGGTACACAT* |
| *Gdf15* | *5’-GAGCTACGGGGTCGCTTC* | *5’-GAGTCCTCTCGGCTCTGGT* |
| *Cdsn* | *5’-CCATCACCTCTGTCCAGAAACC* | *5’-TGTCCTTGGTGAAGTAGCCCAC* |
| *Smpdl3b* | *5’-TGTGGAACGCTTGACCAACCTC* | *5’-TGGGAACTGGTTCTTAGGGTGG* |
| *Stc2* | *5’-CAACGCTGGAAAATTCGATGCCC* | *5’-CTGGACACTTCCTGCTGATGCA* |
| *Masp1* | *5’-CCTTCAAAGACCAAGTGCTCGTC* | *5’-ACTCCATGCACCGTCCTTCAGA* |
